# Metal-Organic Frameworks for Bioimaging: Strategies and Challenges

**DOI:** 10.7150/ntno.63458

**Published:** 2022-01-01

**Authors:** Yanfei Liu, Ting Jiang, Zhenbao Liu

**Affiliations:** 1 Department of Pharmaceutics, Xiangya School of Pharmaceutical Sciences, Central South University, Changsha, 410013, Hunan Province, P. R. China.; 2 Department of Pharmaceutical Engineering, College of Chemistry and Chemical Engineering, Central South University, Changsha, 410083, Hunan Province, P. R. China.; 3 Molecular Imaging Research Center of Central South University, Changsha 410008, Hunan, P. R. China.

**Keywords:** Metal-organic frameworks, Optical bioimaging, Magnetic resonance imaging (MRI), Computed tomography

## Abstract

Metal-organic frameworks (MOFs), composited with metal ions and organic linkers, have become promising candidates in the biomedical field own to their unique properties, such as high surface area, pore-volume, tunable pore size, and versatile functionalities. In this review, we introduce and summarize the synthesis and characterization methods of MOFs, and their bioimaging applications, including optical bioimaging, magnetic resonance imaging (MRI), computed tomography (CT), and multi-mode. Furthermore, their bioimaging strategies, remaining challenges and future directions are discussed and proposed. This review provides valuable references for the designing of molecular bioimaging probes based on MOFs.

## Introduction

Metal-organic framework (MOF), a kind of coordination polymer, has drawn widespread attention since its first put forward as polymeric frameworks consisting of 3D linked rod-like segments by Robson *et al.* in 1989 1. Later, Yaghi *et al.* 2 reported a 2D coordination compound synthesized from a rigid organic ligand, trimesic acid (BTC), and a transition metal Co, and named it MOF in 1995. It was the first time that the concept of the metal-organic framework has been formally proposed. Over the next two decades, as an independent branch of coordination chemistry, MOF families grew at an astonishing rate. Recently, MOFs have been widely exploited in various aspects, such as storage of materials 3, separation 4-6, purification 7, catalysis 8-13, sensing 14-19, bio-imaging 20, 21, energy storage 22-27, drug delivery 28, 29, etc. MOFs are particularly promising for biomedical candidates in biological sensing and imaging due to their quenching performance and luminescence properties 30.

Since Yaghi *et al.* 2 improved the apparent surface area, pore-volume and stability of MOF-5 31, the biological performance of MOF had significantly been optimized, and the scope of the application had been extensively developed. Compared with traditional materials, MOFs are equipped with many properties, such as biodegradability, biocompatible, high loading efficiency, tunable pore sizes, large specific surface area, topological diversity, easy functionalization, etc. 32 Besides, the large pores, high porosity and large specific surface area of MOFs are all beneficial to improve loading efficiency of the guest molecule. Easy to functionalize, good biocompatibility, water solubility and degradability can improve utilization and recyclability of the drug in the body. Furthermore, forces, such as hydrogen bond effect, Van der Waals forces, π-π stacking between aromatic rings exist in the MOFs 33, 34.

MOFs have been used as novel probes in the field of biosensing. In 2002, Wang *et al.* 35 designed the first sensor involving MOFs. Asefa and coworkers 36 firstly prepared biosensors based on MOF in 2008. Since then, MOFs have attracted great interest as biosensing materials. For example, the most widely used optical sensor, fluorescence, can be turned by the metal, ligand units 37, or the guest molecules 38. Simultaneously, MOF possesses adjustable size, low toxicity, and biodegradability, which provides possibilities for applications *in vivo*.

Especially, benefiting from quenching and intrinsic fluorescence properties of MOF, it has been developed as a fluorescence quencher of dye-labeled aptamers, which enabled detection of various biomolecules, including biomacromolecule (nucleic acid, enzyme-activity/proteins) and small biomolecules (dopamine, amino acid, glucose, and H_2_O_2_) by a fluorescence “turn-on” progress 39. Additionally, through a reasonable design strategy, MOFs can be integrated with intrinsic fluorescence properties, which can be affected by biomolecules and provide a light source for optical imaging. When the MOF is doped with Au 40, Mn^2+^ 41, Gd^3+^ 42, SPIO, Fe_3_O_4_ 43, Fe^3+^/Fe^2+^ 44, fluorescent dyes, upconversion nanomaterials 45 (NaYF4: Yb/Er, Gd^3+^ and Tm^3+^), quantum dots as well as other materials (graphene, dots, nanotubes) 34, MOF will equip with the function of imaging.

In recent years, the emerging functional MOFs have shown significant advantages in the field of bioimaging. Although the previous applications and challenges have been well summarized and presented, these applications are also rapidly updated with the rapid development of MOFs 46-48. Therefore, in this review, the synthesis methods of MOFs and their unique properties, such as rational aperture design, diversity, easy to functionalize, high BET/Langmuir surface area etc., have been summarized. Meanwhile, the research progress in bioimaging applications related to MOF, the preparation of MOF probes, and its detection mechanisms are also reviewed. Furthermore, the challenges and future development directions in this field are further discussed. We hope this review will arouse broad interest and inspire readers to rationally design more biological applications based on the unique properties of MOF.

## Synthesis methods and characterization of MOF

### Synthesis methods of NMOF

**Figure 1:**
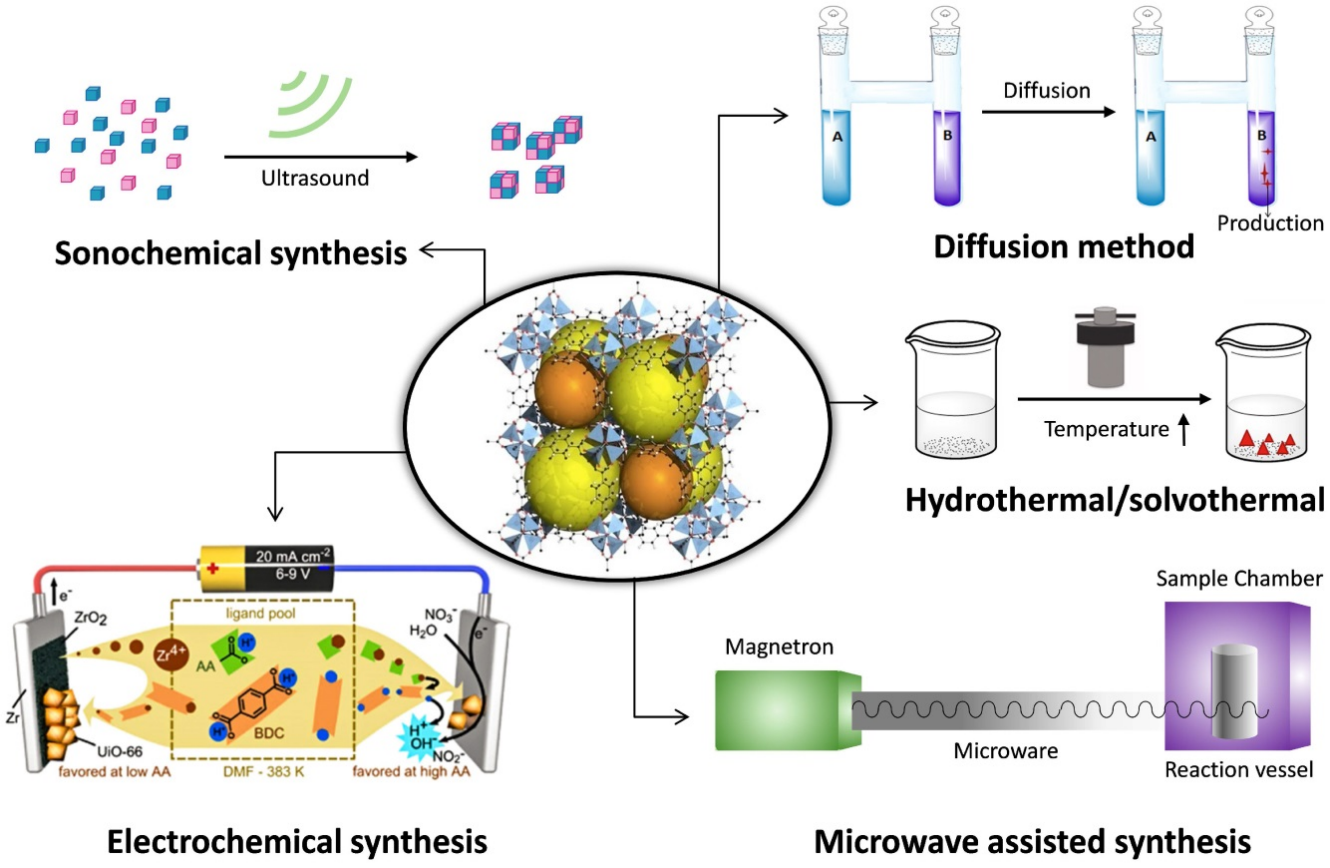
The main synthesis methods of MOFs.

Particle size is a crucial parameter since it determines the physical and chemical properties of the particles 49. Preparation of stable, monodispersed, and nanoscale nanoparticles is necessary. Up until now, various synthesis methods of NMOF have been developed, and the methods are summarized as follows (**Fig. 1**).

#### Hydrothermal/solvothermal

Under the reaction conditions of high temperature (60 and 300 °C) and high pressure, the insoluble reactants at standard temperature and pressure are placed together with solvent and dissolved in a sealed stainless steel reaction vessel lined with polytetrafluoroethylene. And the product is crystallized after reducing the temperature to a certain degree of saturation. These characteristics of MOFs can be adjusted by changing certain experimental parameters, including reaction temperature, reaction time, solvent type, surfactant type, and precursor type. Typically, the ZIF-n family, such as ZIF-8 (40 nm), is primarily synthesized using the solvothermal method 50. This method is also suitable for the MIL-n family, such as flexible porous MIL-89 (50-100 nm), MIL-88A (150 nm), MIL-53 (350 nm), inflexible porous MIL-100 (200 nm), MIL-101-NH_2_ (120 nm), etc. The method owns short synthesis time and it solves the problem of the insolubility of the precursor. The solvents used in the synthesis with different functional groups, polarities, dielectric constants, boiling points and viscosities, can significantly increase the diversity of the synthetic routes and product structures.

#### Microwave-assisted synthesis

Microwave-assisted MOF synthesis yields high-quality MOF crystals with reaction times from 5 seconds to 2.5 minutes, whereas conventional solvothermal and hydrothermal processes require hours or even days. In addition, the MOF material provided by the microwave-assisted method has a uniform crystal size and a well-defined shape, and the size can be adapted to a wide range of applications by controlling the reaction conditions. Typical materials include IRMOF-n (n=1, 2, 3) (100 nm) 51, dual hierarchical porous structure MIL-101(Cr) (22 nm). In this method, two-particle sizes (50 nm, 100 nm) of MIL-101 (Cr) were synthesized by Khan 52 and Zhao 53*,* respectively. Recently, Feng *et al.* 54 successfully synthesized nanoscale medi-MOF-1 with shape, controlled size and graded porosity by microwave heating and growth regulation. Microwave heating retains short reaction times, first kinetics of growing and crystal nucleation, as well as high yields of desirable products that are isolated with no or few secondary products. Advantages also include morphology control, phase selectivity, high efficiency, particle size reduction, etc.

#### Sonochemical synthesis

The mixed solution with a certain proportion of metal salt, organic ligand and an organic solvent is placed in the reaction bottle, using ultrasonic equipment to control the appropriate temperature and power. The violent reaction environment causes the molecules to collide quickly, which accelerates the reaction rate. For instance, microporous flexible MIL-100 (Fe) 55 and MIL-53 (Fe) 56 and Cu_3_(BTC)_2_ (10-200 nm) 57 have been obtained by the sonochemical method. Sonochemical synthesis possesses the advantages of rapidity, facility and etc. Compared with the solvothermal reaction, the reaction time is greatly reduced, and the heating and cooling rates are increased.

#### Electrochemical synthesis

The principle is that the metal ions are first obtained by in-situ anodization and dispersed into a solution containing organic ligands and an electrolyte. The organic ligands are deprotonated and combined with metal ions to form a MOF material. HKUST-1 was the first MOF that was synthesized by the electrochemical synthesis in 2005. Later, MIL-100 (Fe) 58 was prepared by electrochemical deposition under high pressure and high temperature. Electrochemical synthesis is superior environmentally friendly, easy operation and highly efficient synthesis compared with other synthetic methods. Furthermore, it is an effective route for the generation of heterogeneous multiphasic and multilayered MOF layers. However, the limitation of anodic electrodeposition remains.

#### Diffusion method

The diffusion method can be generally divided into three parts: gas-phase diffusion, liquid phase diffusion, and gel diffusion. The gas-phase diffusion method refers to the fact that the two solvents have different choices for the solubility of the target compound and the two solvents have a certain mutual solubility. When the solubility of the solvent diffuses to a solvent with high solubility, the solubility of the compound is lowered, forcing it to continue to crystallize. In the liquid phase diffusion method, a ligand and a metal salt are dissolved in two different solvents. One solution as a buffer was mixed with another solution and chemically reacted. Consequently, crystals may produce near the solution interface. In the gel diffusion method, a ligand is formulated in a gel, agar, or gelatin while a metal salt solution is placed on the gel, and the crystal begins to form when the two components slowly diffuse to the interface. The synthesis conditions are mild, and a high-quality single crystal can be obtained. However, it takes a week to several weeks or a few months.

#### Solvent volatilization method

The appropriate metal salt and organic ligand are selected and dissolved in a suitable solvent, and the polymer crystals are prepared by slowly volatilezing to cause a chemical reaction. The synthesis device of the solvent volatilization method is simple with mild working conditions and highly pure crystal. However, the synthesis takes a long time, and it is necessary to select a suitable solvent.

Besides, the synthesis methods of MOFs also include seed-mediated method 59, post-modification method 60, *in situ* diffusion synthesis 61, microwave-assisted coprecipitation 62, etc. It is worth noting that the synthesis will be affected by many factors, including metal center, organic ligand, reaction solvent, synthesis temperature, pH value of reaction solution, reactant ratio counterion, etc. We need to choose the appropriate method based on the existing experimental conditions and the demand for MOF quality and size.

### Characterization of MOF

Every step of the synthesis of MOF needs to be proved, such as the morphology, pore size, specific surface area, and particle size. In this section, the main methods of characterization have been summarized in **Table 1**.

## MOFs for Bioimaging

The image modes can be divided into two categories: (1) the image providing structural information, such as magnetic resonance imaging (MRI), computed tomography (CT), and ultrasound; (2) the image providing functional and molecular information, such as optical imaging, PET, SPECT, and magnetic resonance spectroscopy. Among them, MRI, CT, and optical imaging are the most common imaging methods. This section will focus on the imaging methods of the above three aspects. Of course, other imaging methods will also be involved.

### Single-modality imaging

#### Optical bioimaging

To date, MOF-based optical imaging has become a hot topic 63. Optical imaging (mainly fluorescence imaging) includes the following aspects: (1) Fluorescent reagent doping-based “turn on” mechanism. Here, MOF is primarily used as a fluorescence quenching platform for fluorescein/dye-labeled probes and the fluorescence will “turn on” due to the binding of probes with the target molecules; (2) Non-fluorescent reagent doped optical imaging, including up-conversion fluorescence imaging and NIR PersL imaging; (3) Undoped optical imaging, in which, the MOF itself is involved in the illumination for imaging; usually, the illuminator has various forms, and the mechanisms are complicated.

##### Fluorescent reagent doping-based “turn on” mechanisms

In this part, most of them are fluorescence imaging based on fluorescent dye reagent doping. Common fluorescent doping reagents include Cathepsin B (CaB), 5-carboxyl fluorescein (5-FAM), TAMRA, rhodamine B isothiocyanate (RBITC), etc. MOF mainly acts as a quenching platform when the target material appears. The fluorescence quenching by the MOF will “turn on”.

Cathepsin B (CaB), abundantly found in the lysosome and one of the cysteine endopeptidases, plays a crucial role in the regulation of cellular metabolism 64. Interestingly, in Shen's group, a core-shell nanoparticle, Au-Cy3P@ZIF-8, had been designed with enzyme and pH dual-recognition switch for localized and high specificity fluorescence imaging of lysosomal CaB in living cells 65. Under the acidic environment, the ZIF-8 shell of the nanoprobes could be disassembled and then the CaB recognized a cleavage site, exposed substrate peptide between arginine and glycine. Due to the departure of a Cy3-labeled, the fluorescence was turned on. Similarly, Liu *et al.* 60 designed a CPC@MOF probe with chlorine e6 (Ce6)-labeled CaB substrate peptide, camptothecin and HOOC-PEG-FA modified MIL-101-NH_2_ using the same mechanism, and MOF was used as a fluorescence quenching platform.

In another study, in 2018, Gao *et al.* 66 obtained a multifunctional system, UIO-66-NH_2_-FA-5-FAM/5-FU, for cancer treatment and fluorescence imaging. After the target drug, 5-FU was adsorbed on MOF, the fluorescence imaging agent 5-carboxyl fluorescein (5-FAM) and the targeted reagent folic acid (FA) were covalently grafted. Predictably, this system displayed good fluorescence imaging *in vitro* based on 5-FAM and due to the targeting effect of folic acid, fluorescence can be enhanced and can target liver cancer cells HePG-2. The difference from the previous two examples is that the imaging system uses FA as a targeting agent to enhance the imaging effect.

**Figure 2:**
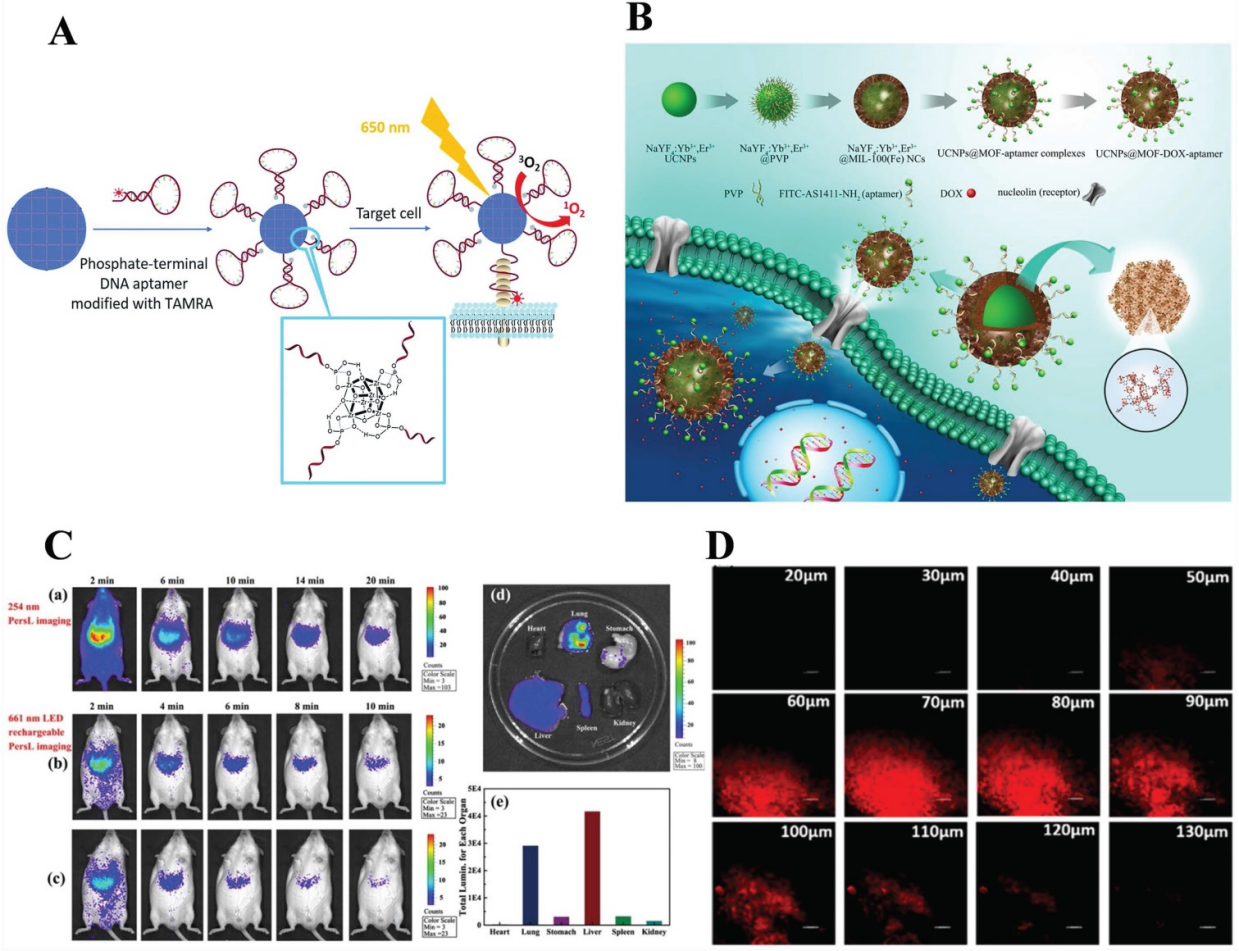
** A) Illustration of phosphate-terminal DNA aptamer conjugation to a Zr-MOF nanoparticle quencher for target-induced imaging and photodynamic therapy.** Reproduced with permission. 67 Copyright 2018, Royal Society of Chemistry. **B)** Schematic illustration of the synthetic procedure, drug loading and receptor-mediated endocytosis pathway of the targeted UCNPs@MOF core-shell NCs. The insets have expanded the view of the porous cavities of MIL-100 (Fe) and part coordination structure, respectively. Reproduced with permission. 70 Copyright 2015, Science. **C)** *In vivo* NIR PersL imaging. a) Mice were intravenously injected with ZGGO@ZIF-8 (0.2 mL, 1 mg/mL in PBS) and radiated at 254 nm for 5 min b) 661 nm LED recharging for 2 min and c) 10 min apart. d) *Ex vivo* NIR PersL imaging. e) Total luminescence of main organs of mice after 24 h of administration. Reproduced with permission. 74 Copyright 2019, American Chemical Society.** D)** Depth fluorescence images of probe 1 (100 µg/mL) in rat liver tissue. The change of fluorescence intensity with scan depth was determined by spectral confocal multi-photon microscopy. Reproduced with permission. 77 Copyright 2019, American Chemical Society.

Also using targeted mediated imaging, Liu *et al.* 67 revealed Zr-based MOF nanoparticles modified with DNA aptamers through strong coordination between zirconium and phosphate for photodynamic therapy and target-induced imaging. Note here that, target-induced imaging is achieved based on the structural change of the aptamer after binding with the target; not only that, the conjugation of Zr-MOF with DNA aptamer results in fluorescence quenching of TAMRA (fluorescent dyes), then target-induced imaging is achieved (Figure 2A).

MOF-based living cells pH imaging in a broad range is a problem to be solved. Chen *et al.* 68 constructed a valid dual-emission fluorescent MOF nanocomposite probe (RB-PCN) for broad-range (pH=1-11) and sensitive pH fluorescence imaging. RB-PCN was prepared with DBI-PEG-NH_2_ functionalized Fe_3_O_4_ as the core and Zr-MOF as a shell and then further reacted with rhodamine B isothiocyanate (RBITC). RB-PCN exhibited two emissions at 641 nm for PCN-224 in response to the alkaline range (red) and at 575 nm for RBITC in response to the acidic range (green) under the single-excitation. In essence, the system was also a fluorescence turn-on strategy caused by the pH-responsive property of the fluorescence reagent RBITC.

##### Non-fluorescent reagent doped optical imaging

Doping fluorescent reagents in MOF is a common fluorescence imaging strategy. In addition, non-fluorescent reagents, such as up-conversion materials and PersL nanoparticles, can also be doped in MOF for fluorescence imaging.

***Up-conversion fluorescence imaging:***

Contrary to ordinary down-conversion illumineting mechanisms, upconversion nanoparticles (UCNPs) used the anti-Stokes effect. It is favored by researchers due to its ability to convert near-infrared radiation into visible light and the properties of high fluorescence efficiency, good stability, and high resolution 69. The introduction of up-conversion luminescent particles also gives the MOF a new development direction.

Deng* et al.* 70 obtained core-shell nanocarriers, UCNPs@MOF-DOX-AS1411, with MIL-100 (Fe) as shell and β-NaYF4: Yb^3+^/Er^3+^ (UCNPs) as core via a two-step approach. The mechanism was that after UCNPs equipped with the aptamers, AS1411, were targeted to the tumor cell surface, nanocarriers exhibit satisfactory up-conversion green emission UC luminescent imaging when exposed to 980 nm laser. (Figure 2B) Similarly, Chowdhuri *et al.* 71 synthesized nanoplatform (UCNPs@ZIF-8/FA) with folate acid (FA) packed by a one-post method for targeting, fluorescence imaging, and pH-responsive drug release. In this research, ZIF-8 with folic acid encapsulated was directly coated on UCNPs (NaYF_4_: Er^3+^, Yb^3+^) to form a monodispersed core-shell structured nanocomposite.

Compared with the former, their work was based on MOF, both using the up-conversion particles NaYF_4_: Er^3+^, Yb^3+^ as optical imaging elements and had higher resolution images. Both of them were core-shell structures and took pH-sensitive MOFs as the shell. Although they were receptor-mediated endocytosis, there are differences in the types of target ligands, the former used AS1411 and the latter employed FA.

***NIR PersL imaging:***

Persistent luminescence (PersL) is a unique optical phenomenon wherein light is able to last for hours or seconds after absent the excitation source 72. Up to now, near-infrared (NIR) PersL nanoparticles (PLNPs) have aroused curiosity among researchers since they can serve as optical probes bioimaging systems with preponderances of low irradiation damage, auto-fluorescence-free, and deep tissue penetration 73. Remind that this principle of optical imaging is different from ordinary fluorescent or phosphorescent.

Recently, a multifunctional nanoplatforms, ZGGO@ZIF-8-DOX, which took chromium (Cr)-doped zinc gallogermanate (ZGGO) NIR PLNPs as a core and ZIF-8 as a shell for dual functionalities of pH drug delivery (LC = 93.2%) and auto-fluorescence-free NIR PersL imaging had been reported 74 (Figure 2C). Most importantly, the PL phenomenon in ZGGO NP was attributed to the ^2^E→^4^A_2_ transition of Cr^3+^. Compared with other imaging methods, the resolution of this platform was higher and could achieve deep tissue penetration. However, the combination of PLNPs and MOF was currently in infancy. Even so, NIR PersL imaging as a special imaging method provided a new idea for optical imaging systems. Compared to the previous fluorescent reagent doping, non-fluorescent reagents doped optical imaging is mainly based on the mechanism of illumination under specific near-infrared light (NIR).

##### Undoped optical imaging

***Single-photon fluorescence bioimaging:***

Compared to the imaging mechanism involved in fluorescent reagents, the imaging involved in non-fluorescent reagents is mainly based on the reaction between MOF and target molecules leading to light-on. For example, peroxynitrite (ONOO^-^), a member of the ROS, while an excessive concentration exists in the organism causes diseases such as diabetes 75. Mostakim SK and coworkers 76 synthesized boronic acid-functionalized Hafnium (Hf)-based MOF, Hf-UiO-66-B(OH)_2_, as a fluorescent turn-on probe for selective sensing and imaging of ONOO^-^. The fluorescent turn-on reaction of the probe to peroxynitrite could be attributed to the oxidative cleavage of the boronic acid group to the corresponding hydroxyl group.

***Two-photon fluorescence bioimaging:***

Single-photon excitation leads to a low signal-to-noise ratio and photobleaching problems. By contrast, two photons fluorescent probes exhibit effective intracellular deep tissue imaging. Yang's group developed a two-photon metal-organic framework (TP-MOF) employing click chemistry for depth fluorescence bioimaging with a penetration of 130 μm in rat liver tissue 77. (Figure 2D) Furthermore, TP organic moiety presented selectivity, photostability, biocompatibility, as well as fluorescence responsive properties. Specifically, unlike other optical imaging, this fluorescence comes from the MOF itself, which acts as the light source in which the components of the MOF participate in the reaction and emit an optical signal under external stimulation. This unique optical signal comes from a specific design of MOF. Significantly, this molecular design concept can be extended to other MOF-based bioimaging, laying a foundation for further research.

#### Magnetic resonance imaging (MRI)

Magnetic resonance imaging (MRI) distinguishes abnormalities in tissues due to changes in nuclear relaxation rate and/or water density through variation in NMR water proton signals. MRI is widely used in clinical imaging because of its non-invasiveness, high spatial resolution, and deep penetration, which can diagnose diseases by changing the longitudinal (T1) and transverse (T2) relaxation rates of water protons. A contrast agent is an imaging enhancement reagent used to shorten imaging time and improve imaging contrast and sharpness. MRI contrast agents are divided into three types: (1) Paramagnetic substances (Gd, Mn, Fe), affects surrounding water molecules and shortens T1 time; (2) Ferromagnetic substances (Fe-Co); (3) Superparamagnetic substances (SPIO, Fe_3_O_4_, Fe^3+^, Fe^2+^), by interfering with the local magnetic field of the human body, it shortens the T_2_ time, the lesion shows a high signal, while the normal tissue shows a low signal. Typically, high paramagnetic metal ion compounds such as Gd^3+^, Mn^2+,^ and Fe^3+^/Fe^ 2+^ are widely used as nuclear magnetic resonance agents. Here, we introduce MRI contrast agents using metal MOFs.

##### Paramagnetic substances (Gd-based/Mn-based)

As an excellent MRI contrast agent, Gd has been fully recognized in imaging due to it can shorten the T1 time and achieve better imaging results. Hatakeyama *et al.* 42 synthesized nanoscale Gd-MOF for PDT and MRI with a high relaxation rate of 40.8 mM^-1^·s^-1^ due to the enhanced rotation-related time and the reduced distance between the proton of Gd^3+^ and hydrogen atoms, also, the increased diffusion time of H_2_O. In addition, McLeod and coworkers incorporate Gd(III) complexes into Zr-MOFs (nano NU-1000, micro NU-1000, NU-901) through solvent-assisted ligand incorporation (SALI) and study the effect of pore shape and particle size on proton relaxation. The results show that Gd nano NU-1000 achieved the best proton relaxivity of 26±1 mM^-1^s^-1^ at 1.4 T 78.

Coincidentally, also combined with porphyrins, Zhang *et al.* 79 firstly developed nanoscale Mn-porphyrin MOF probe, NMOF-SNO, with biocompatible Zr^4+^ ions as the coordination center, Mn-porphyrin as a bridging ligand, and S-nitrosothiol (SNO) functionalized for MRI-guided nitric oxide (NO) detection and synergistic photothermal therapy (PTT). NMOF provided a high molar extinction coefficient, strong T_1_-weighted MR contrast, and adequate photothermal stability after Mn^2+^ inserting into the porphyrin ring successfully.

**Figure 3:**
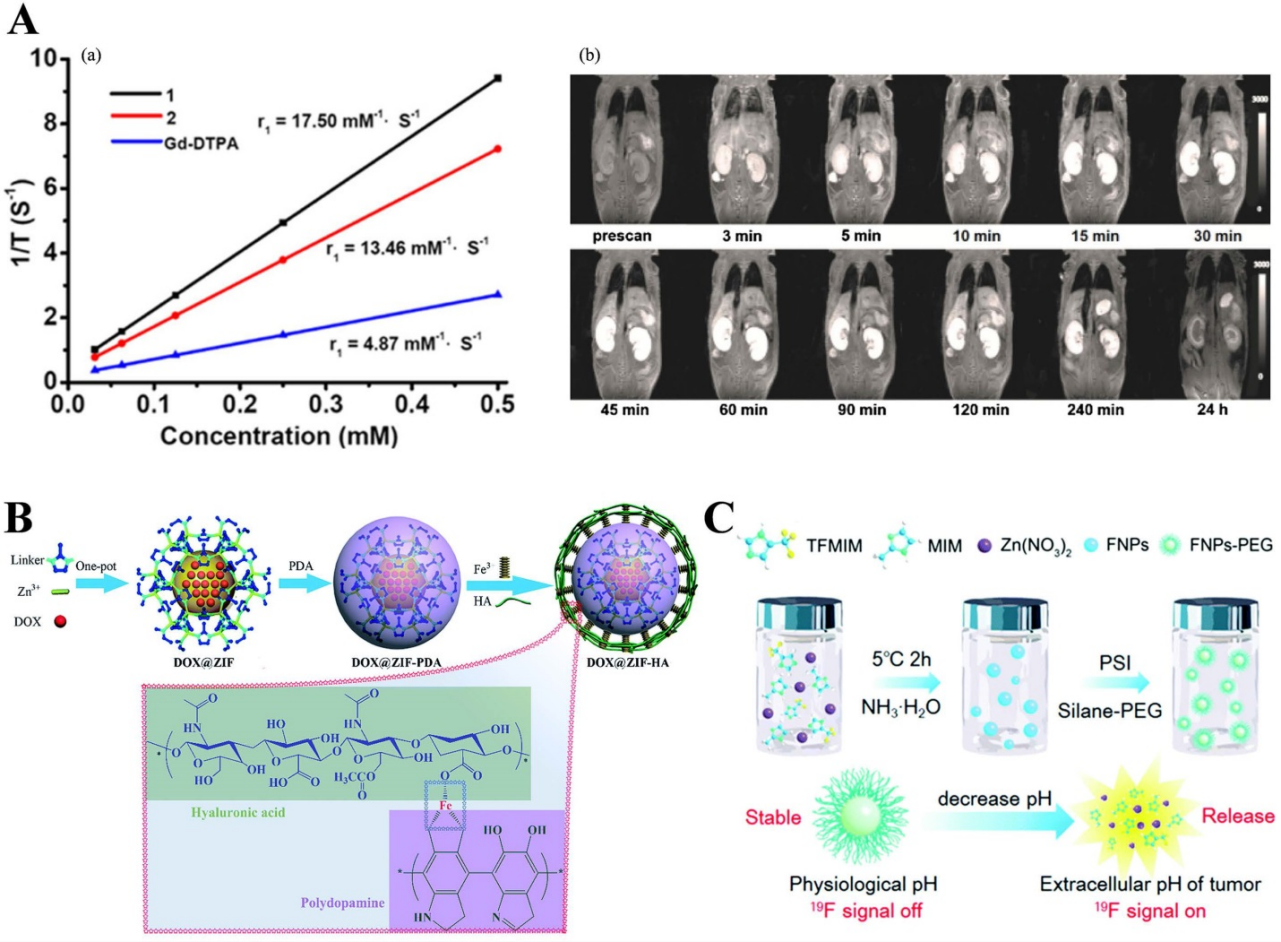
** A)** (a) 1/T1 plot of MOFs 1, 2 and Gd-DTPA concentration. (b) Dynamic MR signal intensity study of a normal kidney after intravenous injection of MOF 1. Reproduced with permission. 80 Copyright 2017, American Chemical Society.** B)** Schematic diagram for the preparation of DOX@ZIF-HA and the Fe^3+^-mediated coordination interaction between HA and PDA. Reproduced with permission. 82 Copyright 2018, Royal Society of Chemistry. **C)** Schematic illustration for the application and preparation of ^19^F MRI probes. Reproduced with permission. 85 Copyright 2018, Royal Society of Chemistry.

Besides, Qin *et al.* 80 designed and synthesized two water-stable 3D Mn-based MOF (**1**) and Gd-based MOF (**2**) as high-efficiency contrast agents for MRI. Furthermore, *in vitro* MRI demonstrated that the relaxation r_1_ of **1** and **2** was 17.50 mM^-1^·S^-1^and 13.46 mM^-1^·S^-1^, respectively, which are higher than the r_1_ (4.87 mM^-1^·S^-1^) of similar Gd-DTPA (DTPA = diethylenetriamine pentaacetate). Compared with** 2**, **1** existed lower cytotoxicity and higher r_1_ relaxivity and revealed excellent kidney-positive signal aggrandizement. MOF** 1** was a potential MRI candidate (Figure 3A).

Actually, the principle of action is the same whether it is Gd or Mn: shortening the T1 relaxation time. In addition to Gd^3+^ and Mn^2+^, more other types of MOFs are also used as contrast agents.

##### Ferromagnetic substances (Fe-based)

In addition, Fe-based MOF is also gaining attention. In 2018, Zhang *et al.* 44 designed a Fe-based nanocomposite, AuNS@MOF-ZD_2_, with a TNBC-targeted peptide (ZD_2_) modified and a gold nanostar (AuNS) coated within MIL-101-NH_2_ (Fe) to achieve T1-weighted MRI and photothermal therapy (PTT) for targeting toward triple-negative breast cancer (TNBC). As a result, nanocomposites present favorable T_1_-weighted magnetic resonance (MR) relaxivity and biocompatibility and efficient photothermal conversion ability (40.5%). *In vitro* and* in vivo* experiments demonstrated satisfactory T1-weighted PTT and MRI performance under a low power density of 808 nm laser, and an excellent hot cathode effect was obtained in TNBC. Another example, Huang *et al.* 81 proposed a multifunctional probe, PPy@MIL-53, by a combination of MIL-53 and polypyrrole (PPy) for MRI-guided PTT of tumors *in vivo*.

Shu* et al.* 82 in 2018 synthesized a hyaluronic acid (HA), a widely reported targeting molecule 83, conjugated ZIF-8 for both targeted and pH-responsive drug delivery and magnetic resonance imaging of prostate cancer PC-3 cells. In this work, DOX@ZIF-8 was formed by the one-pot method, then coated with polydopamine (PDA), sequentially chelated with Fe^3+^, and bound with hyaluronic acid (HA) to form a multifunctional ZIF-8 nanocarrier (DOX@ZIF-HA). Note here that, PDA chelating Fe^3+^ made DOX@ZIF-HA a good contrast agent for MR. More importantly, this was the first time that HA had been combined with ZIF-8 (Figure 3B).

Recently, Xu and others reported Fe-based MOF composed of Fe(III) ions and hematoporphyrin monomethyl ether, loaded with DOX, DOX@FeCPs was used in MR imaging-guided deep tumor sonodynamic chemotherapy combination therapy. T2-weighted MRI *in vivo* was performed on a CT26 tumor-bearing mouse via injecting FeCPs saline and the results demonstrated that FeCPs have passive targeting effects on tumors 84.

Undoubtedly, the superparamagnetic iron ion is the imaging element of MRI in the above cases. The basic principle is to shorten the T_2_ time by interfering with the local magnetic field of the human body. The lesion shows a high signal, while the normal tissue shows a low signal. It's different from Gd^3+^ and Mn^2+^ in the mechanism. Most of the above are ^1^H MRI, which was achieved by affecting the surrounding water molecules.

##### ^19^F MRI

^1^H MRI occupies a dominant position in clinical magnetic resonance technology, but it still faces the challenge because of its strong water background signals *in vivo* and low signal-to-noise ratio. On the contrary, ^19^F MRI has attracted the attention of researchers owing to its high signal-to-noise ratio and lower background signal than ^1^H MRI.

Guo *et al.* 85 used it as a pH-responsive switch to turn on the MRI signal. They fabricated pH-sensitive ^19^F MRI nanoprobes, FNPs-PEG, by partly replacing the 2-methylimidazolate of the ZIF-8, of which the strong ^19^F MRI signal revealed an interesting pH-determined property. Moreover, the contrast agent for pH-responsive (7.4 to 5.5) imaging with low background and high penetration depth. When the surface of PEG was cleaved under acidic conditions, the internal ZIF-8 was cleaved, and the ^19^F-containing TFMIM(4(5)-(trifluoromethyl)imidazole) was released, therefore, resulting in imaging. Interestingly, T_2_ would decrease as the pH increases due to the coordination between zinc and the organic ligand of N-donor in the MOF matrix restricts the movement of ^19^F molecules, resulting in a short T_2_ relaxation time and weak ^19^F signal (Figure 3C). Recently, Zhou and others fabricated Zr-based MOF nanoparticles (UiO-66-F NPs) by a solvothermal method as tumor micro-environment responsive ^19^F MRI contrast agent, enhancing ^19^F MRI signal under acidic conditions but maintain stability under neutral conditions. Importantly, this design concept provides a new concept for future MRI design.

#### Computed tomography (CT)

Computed tomography (CT), one of the most prevalent imaging techniques in the clinic due to its cost-effective, anatomical imaging ability, and wide availability, provides high-resolution 3D tomography anatomical information. In the body, the contrast agent enhances the distinction between organs and tissues of similar density. Nevertheless, the non-specific distribution and fast clearance of the contrast agent *in vivo* limit its clinical application. With the development of nanotechnology, some MOF-based contrast agents have been used to improve CT imaging ability and efficacy.

**Figure 4:**
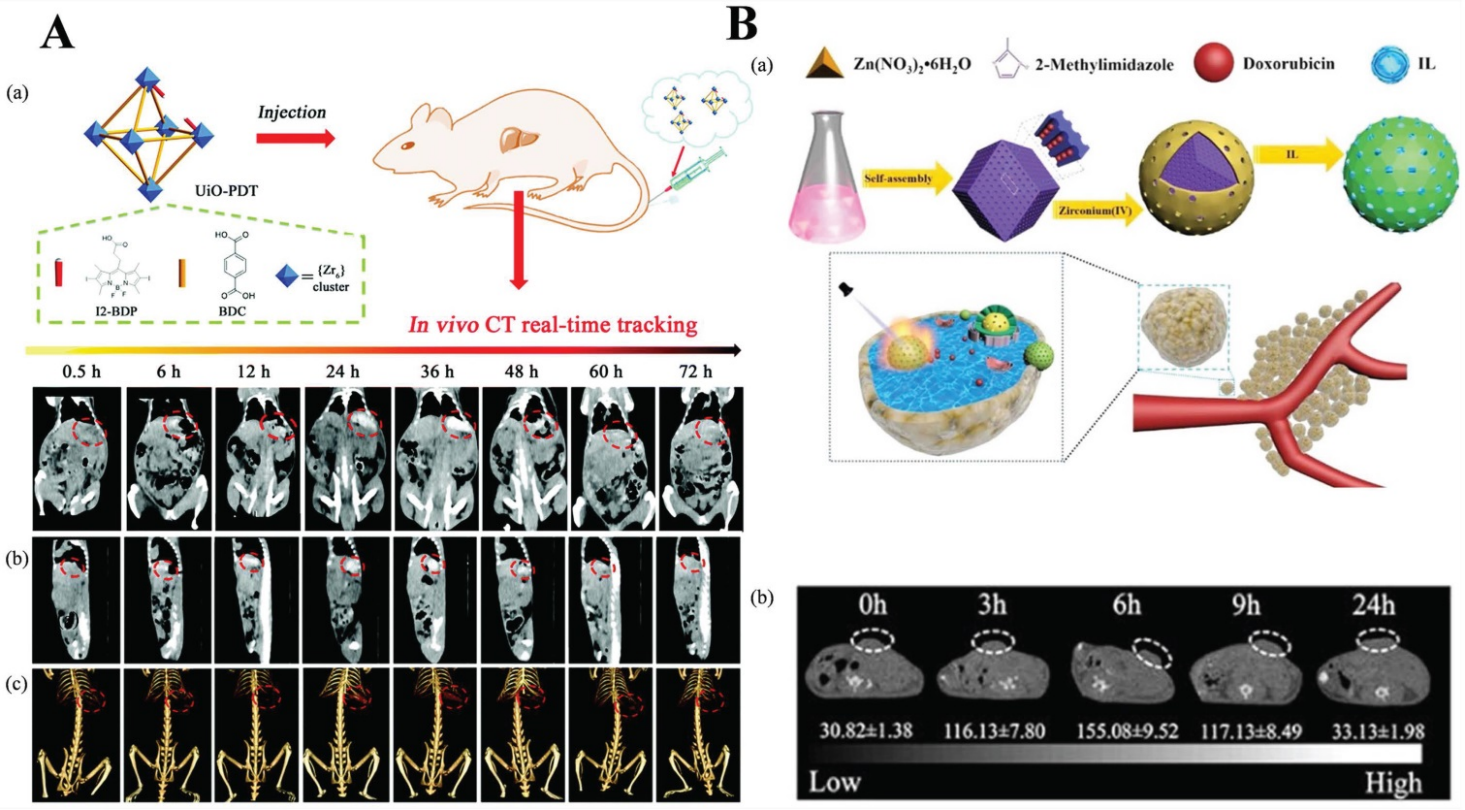
** A)** Schematic illustration of the synthesis of UiO-PDT nanocrystals and their application for *in vivo* X-ray CT imaging and biological studies. Reproduced with permission. 86 Copyright 2017, Royal Society of Chemistry. **B)** Schematic diagram of the fabrication of ZIF-8/DOX@ZrO_2_@IL nanocomposites and application for *in vivo* CT imaging and therapy. Reproduced with permission. 89 Copyright 2019, American Chemical Society.

Zhang* et al.* 86 reported a kind of iodine-boron-dipyrromethene (BODIPY) containing NMOF, UiO-PDT, as CT contrast agents. The results of *in vivo* animal experiments and* in vitro* toxicity analysis showed that there was no apparent toxicity even at a high dose of 100 mg kg^-1^. That is to say, the bioavailability of UiO-PDT was favorable. What's more,* in vivo* CT imaging results showed that UiO-PDT nanoparticles could be preferentially clustered in the tumor site of liver cancer rates. However, no aggregation was observed in the organs and non-surrounding connective tissue. After 24 h, the best CT imaging performance was achieved. All of these results suggest that the UiO-PDT may be a promising candidate for future CT imaging (Figure 4A) 87.

More recently, Robison et al. 20 solvothermal synthesized a bismuth-based MOF (Bi-NU-901) as a contrast agent for X‑ray computed tomography (X-ray CT). Most importantly, *in vivo* research showed that Bi-NU-901 provided ~7 times better contrast intensity compared to Zr-MOF, which possess the same topology, and ~14 times better than a commercial CT contrast agent. Besides, Zhang et al. 88 designed two probes, including DOX@Gd-MOFs-Glu and Yb-MOFs-Glu, for tumor-specific targeting pH-responsive drug delivery and CT imaging. This platform showed good biocompatibility. That means the platform achieved a multifunctional strategy of “three birds with one stone”.

Except for MOF itself as a contrast agent, modifying the surface of the MOF with a contrast agent or encapsulating the contrast agent in the MOF is also a strategy. Su *et al.* used a one-pot method to prepare ZIF-8/DOX, then coated with the contrast agent ZrO_2_ and loaded with an ionic liquid to form a nanocomposite material ZIF-8/DOX@ZrO_2_@IL, which is used for chemo-microwave thermal therapy of tumors. ZrO_2_ coating not only reduced toxic side effects and exhibited biocompatibility but also enhanced the efficacy of CT imaging. It is worth noting that such a MOF complex provided a promising platform for CT imaging (Figure 4B) 89.

### Multimode bioimaging

Generally speaking, due to the inherent limitations of the single-imaging technique, it is unlikely to be used for comprehensive diagnostic information 93. Different imaging modes can often complement each other, which requires the fusion of different imaging modes. As a result, “multimode imaging technology” has developed rapidly in recent years.

**Figure 5:**
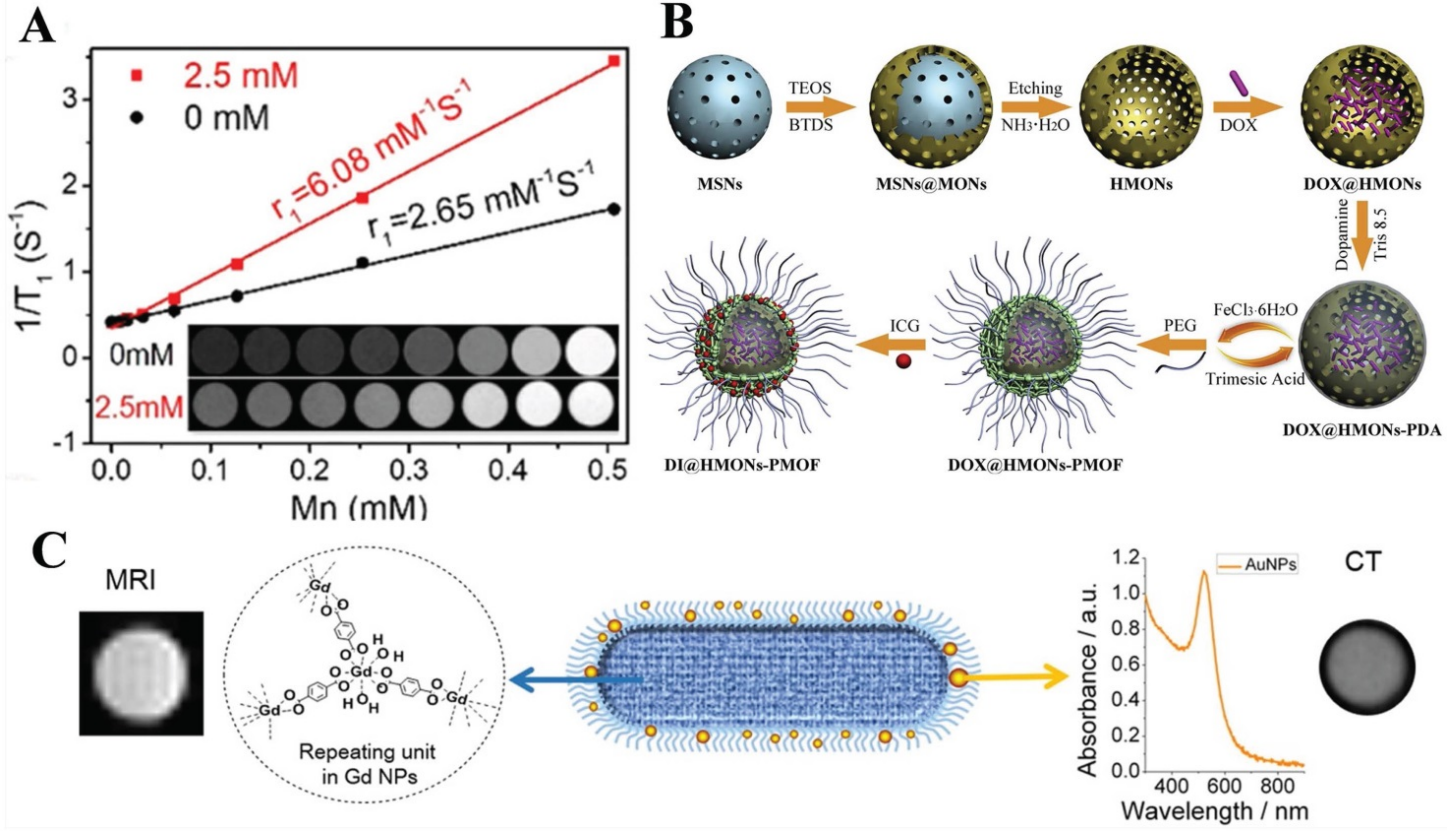
** A)** MOFs t1-weighted imaging with or without glutathione. Reproduced with permission.^41^ Copyright 2019, American Chemical Society. **B)** The fabrication of DOX@HMONs-PMOF. Reproduced with permission. 99 Copyright 2019, Elsevier. **C)** Diagram of GdMOF/AuNPs hybrid nanocomposites apply for MR AND CT imaging. Reproduced with permission. 101 Copyright 2015, American Chemical Society.

**MRI/OI**, MR/fluorescence dual-mode imaging as one of the most common imaging modes in multimode imaging, which has been widely studied 94. Wang *et al.* 95 prepared Gd and Eu Co-doped T_1_-T_2_ dual-mode probes, Eu, Gd-NMOF@SiO_2_, for simultaneously enhancing contrast on T_1_- and T_2_-weighted MRI and fluorescence imaging. In addition, Gao *et al.* 96 designed a Fe-based and smart multifunctional platform, Fe-MIL-53-NH_2_-FA-5-FAM/5-FU, for targeted drug delivery and fluorescence/MR dual-mode imaging. 5-FAM serves as a reagent for fluorescent imaging, while Fe-MIL-53-NH_2_ was used for MRI (T_2_-magnetic response) and encapsulating the drug. Meanwhile, the FA acted as the targeted reagent. *In vitro* cell experiment verifies that the platform possesses better imaging performance and can produce better toxicity to MGC-803 cells with the release of 5-FU. Besides, the same year, Wang* et al.* 97 reported a biodegradable core-shell dual-MOFs, CSD-MOFs, with Prussian blue (PB) as the core and ZIF-8 as the shell for both fluorescence imaging (FI) and MRI dual-mode imaging. Xie *et al.* 98 presented a fresh multi-functional nano-drug carrier UC@mSiO_2_-RB@ZIF-O_2_-DOX-PEGFA (URODF) with pH-responsive chemotherapy. Most importantly, NaYF_4_:Yb/Er@NaYbF_4_:Nd@NaGdF_4_ nanoparticles (UC) were exploited as both MR/upconversion imaging matrices. In addition, Mn(III) sealed MOF with porphyrin as an organic ligand was synthesized by Zhang's team, which reacted with the over-expressed GSH of tumor cells to generate Mn(II) and free porphyrin. With the consumption of GSH, Mn(II)-based MRI and porphyrin-based optical imaging were activated. As shown in Figure 5A, compared with a control group, T1 relaxation rates (r1) increased nearly 2.3 times in the presence of GSH (2.5 mM) 41.

**MRI/PAI**, the combination of MOF and other carrier materials simultaneously, also opens a way for the development of imaging. In Chen's group 99, molecularly organic/inorganic hybridized nano-platforms (HMONs-PMOF) were synthesized by merging with a polydopamine (PDA) interlayer and hollow mesoporous organosilica nanoparticles (HMONs). Benefiting from ICG confined in MOF and the Fe coordinated on PDA, nanocomposites were equipped with photoacoustic (PA) and magnetic resonance (MR) dual-modality imaging. In the process, the two types of MOF play a synergistic role (Figure 5B).

**MRI/CT**, as described in single-mode imaging, CT has excellent performance of imaging, however, due to the similar electron density of CT, it has a poor resolution in soft tissue 100. Whereas MRI has the ability of deep penetration and high spatial resolution, which provides good contrast for soft tissue, but has relatively low sensitivity. Therefore, combining the advantages of several imaging methods may be achieved to provide more accurate and complex diagnostic information. Tian *et al.* 101, for the first time, designed GdMOF-PAA-Au hybrid system as imaging contrast agents for MRI and CT by using poly (acrylic acid), also known as PAA, as a bridge between the AuNPs and Gd-MOF. Note here that, AuNPs provided the ability of CT, while the addition of Gd-MOF was the source of MRI. The results showed that the r_1_ of MRI was 4.9 mM^-1^s^-1^ which is approximately equal to Gd-MOF without modification (r_1_=4.5 mM^-1^s^-1^) and better than Magnevist which is a kind of MRI contrast agent used in clinical. At the same time, experiments have proved that Gd-MOF-PAA-Au hybrid nanocomposites can enhance the contrast of CT imaging even when the Au concentration is as low as 1.66 mg/mL (Figure 5C).

**MRI/PTI**, take advantage of the particular advantages of ultrathin 2D MOF, Li *et al.* 102 reported ultrathin 2D Cu-based MOF nanosheets, Cu-TCPP (TCPP = tetrakis (4-carboxyphenyl) porphyrin), with a facile method of solvothermal for MR/IR thermal imaging and photodynamic therapy (PDT) and photothermal therapy (PTT). As a result, due to the ultrathin properties and the d-d band transition of Cu^2+^, it has excellent NIR capacity and photothermal conversion performance. Meanwhile, the sheet can produce singlet oxygen due to the inherent properties of TCPP. Furthermore, copper is capable of MRI because it has three pairs of unpaired electrons.

***Triple-mode imaging:***

Based on the targeted mediated imaging, the imaging mode gradually changes from dual-modal to three modal 103.

**MRI/OI/PAI**, in the group of Cai 104, MOF@HA@ICG NPs were obtained based on MIL-100 (Fe) for cancer theranostics and multimodal imaging, including PAI, T_2_-weighted MRI, and FL. The result showed a high loading of indocyanine green (ICG, 40%) which was the only NIR organic dye, photostability and strong NIR absorbance. Furthermore, *in vivo* and *in vitro* data demonstrated that the MOF@HA@ICG NPs could be facile uptaken by CD44-positive MCF-7 cells and accumulated in xenograft tumors which were benefit from their targeting capability based on hyaluronic acid (HA).

**Figure 6:**
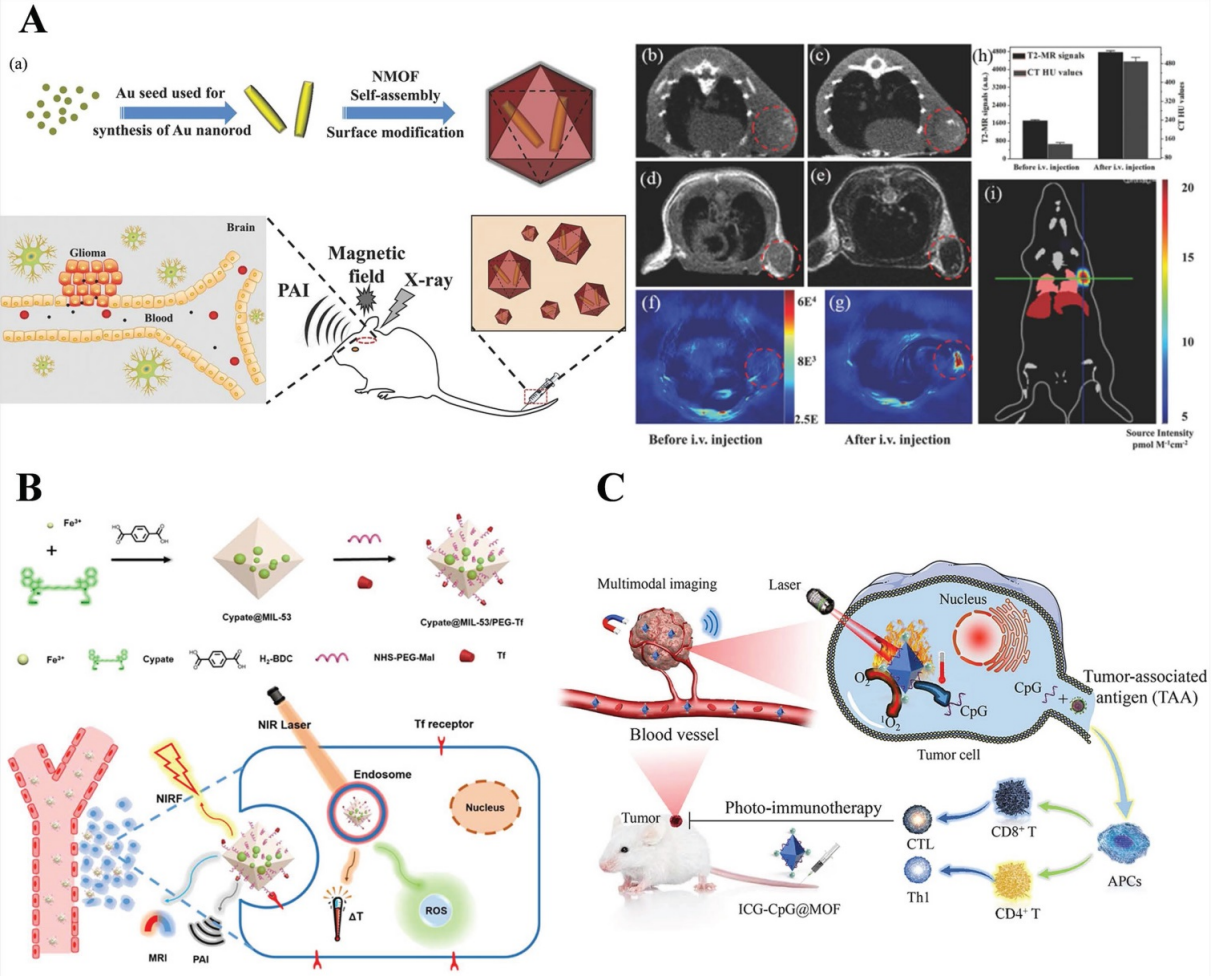
** A)** a) Schematic diagram of the synthesis and biological application of Au@MIL-88(Fe) nanostars. b, c) CT images of mice; d, e) T2-weighted MR images of mice; f, g)* in vivo* PA imaging of tumors in mice; h) quantified MRI and CT signals of tumors from mice; i) bioluminescent imaging of tumor. Reproduced with permission.^105^ Copyright 2017, Wiley. **B)** Schematic illustration of the preparation and its bio-application for multimodal imaging-guided phototherapy of Cypate@MIL-53/PEG-Tf (denoted as “CMNP-Tf”) nanoplatform. Reproduced with permission. 106 Copyright 2019, American Chemical Society. **C)** Schematic diagram of preparation and multimodal imaging-guided photo-immunotherapy of ICG-CpG@MOF. Reproduced with permission. 92 Copyright 2021, Elsevier.

**MRI/CT/PAI**, photoacoustic imaging (PAI), which is based on the photoacoustic effect, is a promising biomedical imaging method with significant spatial resolution and imaging depth and can make up for the deficiency of CT and MRI. Shang* et al.* 105 synthesized a star-shaped core-shell structure nanoprobe, Au@MIL-88 (Fe), multifunctional imaging of glioma, including MRI, CT, and PAI. The results showed that Au@MIL-88 (Fe) provided high contrast and demonstrated negligible cytotoxicity, *in vivo* and* in vitro* experiments showed that Au@MIL-88 (Fe) exhibited a better performance in various diagnostic imaging methods, especially in terms of CT radiological exposure level (Figure 6A).

**NIRF/PAI/MRI**, transferrin (Tf), a tumor-targeting agent, achieves Tf receptor-mediated endocytosis. For targeted precision imaging, Yang* et al.* 106 designed nanoparticles, cypate@MIL-53-Tf nanoparticles (denoted as CMNP-Tf), based on MIL-53 with NIR dye to achieve multimodal-imaging (NIRF/PA/MR) and guided phototherapy. T1-weighted MRI contrast property was ascribed to the Fe^3+^ contribution to the longitudinal relaxivity of MRI and NIRF/PA imaging due to the inherent nature of near-infrared dye (Cypate). Hence, it opened a new way to develop a multifunctional nanoplatform for bio-applications (Figure 6B).

**MRI/OI/PAI/PTI**, Fan et al. synthesized the ICG-CpG@MOF nano-platform with MIL 101-NH_2_ as the core carrier and double-modified with ICG and CpG ODN, which is used for multimodal imaging-guided photo-immunotherapy. Among them, ICG as a donor of photoacoustic and fluorescence imaging applied for photoacoustic, photothermal and fluorescence imaging, and MIL101(Fe) is used for T2-weighted MRI (Figure 6C) 92.

In conclusion, the ultimate goal of bioimaging is to perform ultrasensitive detection of some biomolecules by probes to high resolution, signal stable images. Complementarity between the multimode imaging, cooperate with each other to achieve the ultimate purpose of bioimaging. Considering, only a tiny part of single-mode imaging and multimode imaging are mainly introduced. Other common imaging modes are listed in **Table 3** below.

## Discussion and Prospectives

In this review, a systematic introduction of the MOF-based imaging system was presented, and several common sensors and the mechanisms of sensing were summarized. The imaging models were divided into single modal imaging and multimodal, depending on whether it is for single or multiple imaging. In the single-mode imaging section, some common imaging modes, such as optical imaging, MRI, and CT were highlighted. To the best of our knowledge, the optical imaging section has seldom been classified and described according to the doping situation. For the multimode imaging part, several of the most recent typical imaging modes have been introduced, such as MRI/optical imaging.

The current achievements exhibited the promising future of MOF for bioimaging. However, there are still many challenges that cannot be ignored. For example, MOF as a hybrid material containing metals and organics, biotoxicity and biocompatibility are the biggest challenges limiting the further clinical applications of MOF. Secondly, the poor colloidal stability and solubility of MOFs in biological media greatly limit their application for *in vivo* biological imaging. Thus, green synthetic routes and the “from source design” strategy should be adopted.

As far as bioimaging is concerned, the challenges are mainly in the following aspects: (1) Designing high resolution, signal stability, intelligent targeted and extend fluorescence lifetime imaging systems; (2) Reducing biotoxicity and increases biodegradability. Take MRI as an example, the toxicity of Gd^3+^, Mn^2+^ has limited its widespread clinical use due to their irreversible and tight binding to the serum proteins 159, also, Gd^3+^ may cause deposits in the brain 160. (3) Reducing the amount of contrast agent when maintaining the same effect is needed to decrease the side effects.

## Conclusion

In this review, the unique properties of MOF, such as rational aperture design, diversity, easy to functionalization, high BET/Langmuir surface area, etc., were highlighted, showing the incomparable charm of MOF. Meanwhile, the review also summarizes and discusses the bioimaging applications based on MOF, indicating MOF is a quite promising platform for bioimaging. Biotoxicity and lacking clinical studies are major problems for MOF-based bioapplications. Although MOF is still in its infancy, with the overcome of weaknesses, MOF-based bioimaging platforms are promising in the future.

## Figures and Tables

**Table 1:** Characterization methods of MOF and functionalities

Methods	Abbreviation	Functionalities
Single crystal X-ray diffraction	SCXRD	Characterization of crystal structure
Powder X-ray diffraction	PXRD	Characterization of crystallinity and phase purity
Brunauer-Emmett-Teller	BET	Detection of pore volume, pore size distribution
Dynamic laser scattering	DLS	Particle size distribution
Scanning electron microscopy	SEM	Detecting the surface topography and size distribution of the crystal
Transmission electron microscopy	TEM	Detecting the morphology and thickness of the crystal
Thermogravimetric Analysis	TGA	Thermal stability analysis
UV-Visible Spectroscopy	UV-vis	Structure Identification
^1^H nuclear magnetic resonance	^1^H NMR	Determine the structure of the molecule
Fourier Transform Infrared Spectroscopy	FTIR	Determination of functional groups and chemical structures of organic compounds in crystals
Neutron diffraction	-	Crystal space structure determination, the study of adsorption sites
N_2_ absorption-adsorption spectrometer	-	Specific surface area detection
Barrett-Joyner-Halenda absorption method	BJH	Pore size, Porosity

**Table 2:** Summary of clinical imaging modalities and related nanomaterials as contrast agents

Modality	Measured signals	Resolution	Depth	Related nanomaterials
Magnetic resonance imaging (MRI)	Magnetic field variations	25-100 μm	No limit	SPIO, Fe_3_O_443_, Fe^3+^, Fe^2+^ 44 nanoparticles (T_2_), Mn(II) 41 and Gd(III) 42 doped nanoparticles (T_1_)
Optical imaging	Light	1-3 mm	< 2 mm	Dye-loaded nanoparticles 65, upconversion nanomaterials (NaYF_4_:Yb/Er, Gd^3+^ and Tm^3+^), quantum dots, modified carbon-based materials (grapheme, dots, nanotubes) 34
Computed tomography (X-ray CT)	X-ray	50 μm	No limit	Iron oxide-doped nanomaterials, iodinated nanoparticles, high-Z elements (e.g., Hf and Zr) 90
Single photon emission computed tomography (SPECT)	γ-ray	1-2 mm		Nanomaterials with radioisotopes such as^99m^Tc and ^111^In 91
Positron Emission Tomography (PET)	Positron from radioactive nuclides	1-2 mm		Nanoparticles with radioisotopes such as ^18^F, ^11^C, ^64^Cu, and ^124^I
Photoacoustic imaging (PAI)	Photoacoustic signal	~1 mm	50 mm	Near-infrared dye-loaded (Cypate, indocyanine green) 92, upconversion nanoparticles (UCNPs), carbon nanomaterials, gold nanorods
Fluorescence reflectance imaging (FRI)	Light	< 1 cm	< 1 cm	-
UM/UI	Sound	50 μm	mm to cm	Microbubbles, emulsions, polystyrenebeads
PAM/PAT	-	mm to cm	mm to cm	Gold nanomaterials, carbon nanotubes,dye-loaded nanomaterials

**Table 3:** Bioimaging applications of MOFs

Probes	Imaging agent	MOFs	Imaging applications	Detection objects	Refs.
Hf-NMOF@SiO_2_@PEG	Hf	Zr-UiO	CT	spleen and liver	107
Fe-MIL-53-NH_2_-FA-5-FAM/5-FU	Fe	Fe-MIL-53-NH_2_	OI and MRI	MGC-803/HASMC	96
Rs⊂nMOF-801 R6G⊂nUiO-67	R6G	MOF-801 UiO-67	OI	FL83B HepG2	108
RhB@Al-MOF/5-FU/TCH	RhB	Al-MOF	OI	HASMC/MGC-803	109
MCM@PEG-CO-DOX	CO	MIL-100	MRI and PAI	HCT116	110
MCH NPs		MIL-100	PAI	HeLa	111
TCPC-UiO	Hf	Hf-UiO-66	CT/thermal/photoacoustic	H_2_O_2_	112
Gd/Tm-MOFs@mSiO_2_-FA	Gd	Gd/Tm-MOFs	UCL/MRI	MCF-7	113
cal-TPP@(DCA5-UiO-66)	calcein	UiO-66	OI	MCF-7	114
NUS 27-29 NSs@ZIF-8	NUS 27-29	ZIF-8	OI	HeLa/MCF-7/231/NIH-3T3	115
Fe_3_O_4_/RhB@Al-MOFs	RhB	Al-MOFs	OI	Mg^2+^	116
UiO-68-R6G	UiO-68-R6G	UiO-68-R6G	OI	Hg^2+^	117
UiO-66-NH_2_@N-CNDs	N-CNDs	UiO-66	OI	A549	118
NaLnF_4_@MOF-Ln	Eu^3+^, Tm^3+^/Yb^3+^	NaYF4, MOFs-Y	OI	Hela	45
Fe_3_O_4_@UIO-66-NH_2_/graphdiyne	DOX	Fe_3_O_4_@UIO-66-NH_2_ (FU)	OI	HeLa	34
UiO-66@DOPA-LB	IR-800	UiO-66	OI	4T1	119
PdH-MOF	PdH-MOF	Pd-MOF	PAI	4T1	120
PCN-CuS-FA-ICG	ICG	PCN-224	OI and thermal imaging	MDA-MB-231	121 ^1^
DNA amplifier-MOF		UiO-66	OI	mRNA	122
DNA@Cu-MOF		Cu-MOF	OI	miRNAs	123
FZIF-8/DOX-MIPs	DOX	ZIF-8	OI	MCF-7	124
RhB/Fe_3_O_4_/ZIF-8	RhB	ZIF-8	OI	HePG-2	125
Bismuth-NU901	Bismuth-NU901	Bismuth-NU901	CT		20
ZIF-8/DOX@ZrO_2_@IL	ZrO_2_	ZIF-8	CT		89
Gd-MOFs-Glu/Yb-MOFs-Glu	Gd^3+^/Yb^3+^	Gd-MOFs/Yb-MOFs	MRI/CT	Kidneys, liver/gastrointestinal tract	21
Zn-TCPP@PEG NPs	^99m^Tc	Zn-TCPP	SPECT	Liver, heart, muscle and tumor	91
HUC-PEG	Hf	Hf-UiO-66	CT/photothermal		126
^99m^Tc-PCN-PEG	^99m^Tc	PCN-224	SPECT/CT		90
PCN-222(Mn)	Mn	PCN-222(Mn)	MRI	Kidneys and liver	127
APT-Mn-ZIF-90	Mn	ZIF-90	MRI	MCF-7	128
Fe_3_O_4_-ZIF-8	Fe_3_O_4_	ZIF-8	MRI	Tumor and liver	33
DOX/Fe-G@Z	Fe	ZIF-8	MRI	MCF-7 cells	129
Mn-ZIF-8	Mn	ZIF-8	MRI	U87-MG	130
Fe_3_O_4_@Bio-MOF	Fe_3_O_4_	Fe3O4@Bio-MOF	MRI	MDA-MB-231	131
DOX@PCN@MnO2@PAH	Mn^2+^	PCN-222	MRI	4T1	132
Mn(III)-TCPP	Mn(III)	Mn(III)-TCPP	MRI/OI	4T1	41
MCOPP-Ce6	Mn^2+^, Ce6	Mn_3_[Co(CN)_6_]_2_	MRI/OI	4T1	133
INH-MOF	Fe	Fe-MIL-101-NH_2_	MRI	Lungs	134
Gd-ZMOF	Gd(III)	Gd-ZMOF	MRI	4T1	135
FA-PPSM	Mn^2+^	Zr(IV) porphyrin MOF	MRI	Tumor	136
MIL-Cur@FC	Fe	MIL-88B,	MRI	Liver and tumor	137
Fe_3_O_4_@ALA-Zn MOF	Fe_3_O_4_	ALA-Zn MOF	MRI		138
Hep-(Rap@MOF)@PCL	Fe	(MIL)-101(Fe)	MRI		139
Gd(III)-Functionalized Zr-MOFs	Gd(III)	Zr-MOFs	MRI		78
Au@Fe(BTC)_3_(H_2_O)_6_	Fe	Fe-MOF	MRI	MDA-MB-231	140
CuS@Fe-MOF	Fe	CuS@Fe-MOF	MRI	CT26	141
Fe_3_O_4_@C-PVP@DOX	Fe_3_O_4_	Fe-MOF	MRI		43
DOX@FeCPs	Fe	Fe-HMME	MRI	CT26	84
Fe_3_O_4_-NH_2_@PDA@Au@MIL101-NH_2_	Fe_3_O_4_	MIL 101	MRI	Hela cells	142
MIL-101(Fe)@sor	Fe	MIL-101	MRI	HepG2	143
Mn^2+^&DOX@MOF	Mn^2+^	UiO-66(Zr)-(COOH)_2_	MRI	4 T1	144
UiO-66-F/PPEG NPs	^19^F	UiO-66-F	MRI	4 T1	145
Fe-DOX@Gd-MOF-ICG	Gd, ICG	Gd-MOF	MRI/PAI/PTI	4T1	146
UPFB	UCNPs/Fe^2+^	PCN-224(Fe)	MRI/OI	U14	147
Gd-PDA-Ce6@Gd-MOF	Gd^3+^/PDA	Gd-MOF	MRI/PAI	4T1	148
RhB@Gd-MOFs	Gd^3+^/RhB	Gd-MOFs	MRI/OI	HepG2	149
ICG@Mn/Cu/Zn-MOF@MnO_2_	Mn^2+^/ICG	ZIF-90	MRI/OI/PTI	U87	150
Gd/Tm-PB@ZIF-8/PDA	Gd^3+^	ZIF-8	MRI/OI/PTI	4T1	151
PCN-224(Cu)-GOD@MnO_2_	Mn^2+^/porphyrin	PCN-224	MRI/OI	U14	152
FePt-MOF NCs	Fe/Pt	MIL-101(Fe)	MRI/CT	4T1	153
UCMOFs@D@5	UCNPs	UCMOFs	MRI/OI(UCL)	HeLa	153
ZIF-8/DMPP	Mn^2+^/PDA	ZIF-8	MRI/PAI	PC-3	154
FA-Hf-Mn-NMOF	Hf/Mn^2+^	Mn-TCPP	MRI/CT/PAI	S180	103
CM-MMNPs	Mn^2+^/porphyrin	Zr-TCPP	MRI/OI	HeLa	155
IL@MIL-101(Fe)@BSA-AuNCs	Fe/AuNs	MIL-101(Fe)	MRI/OI	H22	40
Fe/La-MOFs	Fe^3+^/DOX	La-MOFs	MRI/OI	4T1	156
Fe-MOF-5-NH_2_-FA-5-FAM/5-FU	Fe(III)/5-FAM	Fe-MOF	MRI/OI	HepG-2	157
FA-NPMOFs	Gd^3+^/ porphyrin	Gd-MOF	MRI/OI	HepG-2	158

Magnetic resonance imaging (MRI); Photoacoustic imaging (PAI); Polydopamine (PDA); Photoactive tetratopic chlorin (TCPC); Optical bioimaging (OI); Photothermal imaging (PTI).

## References

[1] Hoskins BF, Robson R (1989). Infinite polymeric frameworks consisting of three dimensionally linked rod-like segments. Journal of the American Chemical Society.

[2] Yaghi OM, Li G, Li H (1995). Selective binding and removal of guests in a microporous metal-organic framework. Nature.

[3] Liang CC, Shi ZL, He CT, Tan J, Zhou HD, Zhou HL, Lee Y, Zhang YB (2017). Engineering of Pore Geometry for Ultrahigh Capacity Methane Storage in Mesoporous Metal-Organic Frameworks. J. Am. Chem. Soc.

[4] Zhang X, Chuah CY, Dong P, Cha YH, Bae TH, Song MK (2018). Hierarchically Porous Co-MOF-74 Hollow Nanorods for Enhanced Dynamic CO2 Separation. ACS Appl Mater Interfaces.

[5] Prasetya N, Ladewig BP (2018). New Azo-DMOF-1 MOF as a Photoresponsive Low-Energy CO2 Adsorbent and Its Exceptional CO2/N2 Separation Performance in Mixed Matrix Membranes. ACS Appl Mater Interfaces.

[6] Marti AM, Venna SR, Roth EA, Culp JT, Hopkinson DP (2018). Simple Fabrication Method for Mixed Matrix Membranes with *in situ* MOF Growth for Gas Separation. ACS Appl Mater Interfaces.

[7] Wang L, Zhu H, Shi Y, Ge Y, Feng X, Liu R (2018). Novel catalytic micromotor of porous zeolitic imidazolate framework-67 for precise drug delivery. Nanoscale.

[8] Liu X, Qi W, Wang Y, Lin D, Yang X, Su R (2018). Rational Design of Mimic Multienzyme Systems in Hierarchically Porous Biomimetic Metal-Organic Frameworks. ACS Appl Mater Interfaces.

[9] Salgaonkar M, Nadar SS, Rathod VK (2018). Combi-metal organic framework (Combi-MOF) of alpha-amylase and glucoamylase for one pot starch hydrolysis. Int J Biol Macromol.

[10] Yang JC, Shang Y, Li YH, Cui Y, Yin XB (2018). An "all-in-one" antitumor and anti-recurrence/metastasis nanomedicine with multi-drug co-loading and burst drug release for multi-modality therapy. Chemical Science.

[11] Zhao W, Wan G, Peng C, Sheng H, Wen J, Chen H (2018). Key Single-Atom Electrocatalysis in Metal-Organic Framework (MOF)-Derived Bifunctional Catalysts. ChemSusChem.

[12] Zou MZ, Liu WL, Li CX, Zheng DW, Zeng JY, Gao F (2018). A Multifunctional Biomimetic Nanoplatform for Relieving Hypoxia to Enhance Chemotherapy and Inhibit the PD-1/PD-L1 Axis. Small.

[13] Han SY, Pan DL, Chen H, Bu XB, Gao YX, Gao H (2018). A Methylthio-Functionalized-MOF Photocatalyst with High Performance for Visible-Light-Driven H2 Evolution. Angew Chem Int Ed.

[14] Qu F, Li XN, Lv XX, You JM, Han WL (2019). Highly selective metal-organic framework-based sensor for protamine through photoinduced electron transfer. Journal of Materials Science.

[15] Qin J, Cho M, Lee Y (2019). Ferrocene-Encapsulated Zn Zeolitic Imidazole Framework (ZIF-8) for Optical and Electrochemical Sensing of Amyloid-beta Oligomers and for the Early Diagnosis of Alzheimer's Disease. ACS Appl Mater Interfaces.

[16] Masih D, Chernikova V, Shekhah O, Eddaoudi M, Mohammed OF (2018). Zeolite-like Metal-Organic Framework (MOF) Encaged Pt(II)-Porphyrin for Anion-Selective Sensing. ACS Appl Mater Interfaces.

[17] Lei B, Wang M, Jiang Z, Qi W, Su R, He Z (2018). Constructing Redox-Responsive Metal-Organic Framework Nanocarriers for Anticancer Drug Delivery. ACS Appl Mater Interfaces.

[18] Liu LL, Yu YZ, Zhao XJ, Wang YR, Cheng FY, Zhang MK (2018). A robust Zn(ii)/Na(i)-MOF decorated with [(OAc)2(H2O)2]n(2n-) anions for the luminescence sensing of copper ions based on the inner filter effect. Dalton Trans.

[19] Yang X, Yu YQ, Peng LZ, Lei YM, Chai YQ, Yuan R (2018). Strong Electrochemiluminescence from MOF Accelerator Enriched Quantum Dots for Enhanced Sensing of Trace cTnI. Anal Chem.

[20] Robison L, Zhang L, Drout RJ, Li P, Haney CR, Brikha A (2019). A Bismuth Metal-Organic Framework as a Contrast Agent for X-ray Computed Tomography. ACS Applied Bio Materials.

[21] Zhang H, Shang Y, Li YH, Sun SK, Yin XB (2019). Smart Metal-Organic Framework-Based Nanoplatforms for Imaging-Guided Precise Chemotherapy. ACS Appl Mater Interfaces.

[22] Yang ZL, Tian W, Wang Q, Zhao Y, Zhang YL, Tian Y (2018). Oxygen-Evolving Mesoporous Organosilica Coated Prussian Blue Nanoplatform for Highly Efficient Photodynamic Therapy of Tumors. Adv Sci (Weinh).

[23] Zhang Y, Wang F, Liu C, Wang Z, Kang L, Huang Y (2018). Nanozyme Decorated Metal-Organic Frameworks for Enhanced Photodynamic Therapy. ACS Nano.

[24] Hong XJ, Tang XY, Wei Q, Song CL, Wang SY, Dong RF (2018). Efficient Encapsulation of Small S2-4 Molecules in MOF-Derived Flowerlike Nitrogen-Doped Microporous Carbon Nanosheets for High-Performance Li-S Batteries. ACS Appl Mater Interfaces.

[25] Zou G, Hou H, Ge P, Huang Z, Zhao G, Yin D (2018). Metal-Organic Framework-Derived Materials for Sodium Energy Storage. Small.

[26] Qu C, Liang Z, Jiao Y, Zhao B, Zhu B, Dang D (2018). "One-for-All" Strategy in Fast Energy Storage: Production of Pillared MOF Nanorod-Templated Positive/Negative Electrodes for the Application of High-Performance Hybrid Supercapacitor. Small.

[27] Hu J, Liu C, Liu L, Li Q (2018). Thermal Energy Storage of R1234yf, R1234ze, R134a and R32/MOF-74 Nanofluids: A Molecular Simulation Study. Materials.

[28] Wang S, Chen Y, Wang S, Li P, Mirkin CA, Farha OK (2019). DNA-Functionalized Metal-Organic Framework Nanoparticles for Intracellular Delivery of Proteins. J Am Chem Soc.

[29] Zhao H, Hou S, Zhao X, Liu D (2019). Adsorption and pH-Responsive Release of Tinidazole on Metal-Organic Framework CAU-1. Journal of Chemical & Engineering Data.

[30] Yang P, Men Y, Tian Y, Cao Y, Zhang L, Yao X (2019). Metal-Organic Framework Nanoparticles with Near-Infrared Dye for Multimodal Imaging and Guided Phototherapy. ACS Appl Mater Interfaces.

[31] Li H, Eddaoudi M, O'Keeffe M, Yaghi OM (1999). Design and synthesis of an exceptionally stable and highly porous metal-organic framework. Nature.

[32] Wang Y, Yan JH, Wen NC, Xiong HJ, Cai SD, He QY, Hu YQ, Peng DM, Liu ZB, Liu YF (2020). Metal-organic frameworks for stimuli-responsive drug delivery. Biomaterials.

[33] Lin J, Xin P, An L, Xu Y, Tao C, Tian Q (2019). Fe3O4-ZIF-8 assemblies as pH and glutathione responsive T2-T1 switching magnetic resonance imaging contrast agent for sensitive tumor imaging *in vivo*. Chem Commun (Camb).

[34] Xue Z, Zhu M, Dong Y, Feng T, Chen Z, Feng Y (2019). An integrated targeting drug delivery system based on the hybridization of graphdiyne and MOFs for visualized cancer therapy. Nanoscale.

[35] Wang Z, Jin C-M, Shao T, Li Y-Z, Zhang K-L, Zhang H-T (2002). Syntheses, structures, and luminescence properties of a new family of three-dimensional open-framework lanthanide coordination polymers. Inorganic Chemistry Communications.

[37] Cui Y, Zhu F, Chen B, Qian G (2015). Metal-organic frameworks for luminescence thermometry. Chem Commun (Camb).

[38] Della Rocca J, Liu D, Lin W (2011). Nanoscale metal-organic frameworks for biomedical imaging and drug delivery. Accounts of chemical research.

[39] Wang HS, Li J, Li JY, Wang K, Ding Y, Xia XH (2017). Lanthanide-based metal-organic framework nanosheets with unique fluorescence quenching properties for two-color intracellular adenosine imaging in living cells. NPG Asia Materials.

[40] Ma X, Ren X, Guo X, Fu C, Wu Q, Tan L (2019). Multifunctional iron-based Metal-Organic framework as biodegradable nanozyme for microwave enhancing dynamic therapy. Biomaterials.

[41] Wan SS, Cheng Q, Zeng X, Zhang XZ (2019). A Mn(III)-Sealed Metal-Organic Framework Nanosystem for Redox-Unlocked Tumor Theranostics. ACS Nano.

[42] Hatakeyama W, Sanchez TJ, Rowe MD, Serkova NJ, Liberatore MW, Boyes SG (2011). Synthesis of Gadolinium Nanoscale Metal-Organic Framework with Hydrotropes: Manipulation of Particle Size and Magnetic Resonance Imaging Capability. ACS Appl Mater Interfaces.

[43] Xiang Z, Qi Y, Lu Y, Hu Z, Wang X, Jia W (2020). MOF-derived novel porous Fe3O4@C nanocomposites as smart nanomedical platforms for combined cancer therapy: magnetic-triggered synergistic hyperthermia and chemotherapy. J Mater Chem B.

[44] Zhang L, Liu C, Gao Y, Li Z, Xing J, Ren W (2018). ZD2-Engineered Gold Nanostar@Metal-Organic Framework Nanoprobes for T1 -Weighted Magnetic Resonance Imaging and Photothermal Therapy Specifically Toward Triple-Negative Breast Cancer. Advanced healthcare materials.

[45] Wang D, Zhao C, Gao G, Xu L, Wang G, Zhu P (2019). Multifunctional NaLnF4@MOF-Ln Nanocomposites with Dual-Mode Luminescence for Drug Delivery and Cell Imaging. Nanomaterials (Basel).

[46] Peller M, Böll K, Zimpel A, Wuttke S (2018). Metal-organic framework nanoparticles for magnetic resonance imaging. Inorganic Chemistry Frontiers.

[47] Wang H-S (2017). Metal-organic frameworks for biosensing and bioimaging applications. Coordination Chemistry Reviews.

[48] Yang J, Yang YW (2020). Metal-Organic Frameworks for Biomedical Applications. Small.

[49] Peng X, Manna L, Yang W, Wickham J, Scher E, Kadavanich A (2000). Shape control of CdSe nanocrystals. Nature.

[50] Cravillon J, Munzer S, Lohmeier SJ, Feldhoff A, Huber K, Wiebcke M (2009). Rapid Room-Temperature Synthesis and Characterization of Nanocrystals of a Prototypical Zeolitic Imidazolate Framework. Chemistry of Materials.

[51] Ni Z, Masel RI (2006). Rapid production of metal-organic frameworks via microwave-assisted solvothermal synthesis. J Am Chem Soc.

[52] Kang IJ, Khan NA, Haque E, Jhung SH (2011). Chemical and thermal stability of isotypic metal-organic frameworks: effect of metal ions. Chemistry.

[53] Zhao ZX, Li XM, Huang SS, Xia QB, Li Z (2011). Adsorption and Diffusion of Benzene on Chromium-Based Metal Organic Framework MIL-101 Synthesized by Microwave Irradiation. Industrial & Engineering Chemistry Research.

[54] Feng XD, Wang YF, Muhammad F, Sun FX, Tian YY, Zhu GS (2019). Size, Shape, and Porosity Control of Medi-MOF-1 via Growth Modulation under Microwave Heating. Cryst Growth Des.

[55] Rezaei M, Abbasi A, Varshochian R, Dinarvand R, Jeddi-Tehrani M (2018). NanoMIL-100(Fe) containing docetaxel for breast cancer therapy. Artificial cells, nanomedicine, and biotechnology.

[56] Haque E, Khan NA, Park JH, Jhung SH (2010). Synthesis of a metal-organic framework material, iron terephthalate, by ultrasound, microwave, and conventional electric heating: a kinetic study. Chemistry.

[57] Li ZQ, Qiu LG, Xu T, Wu Y, Wang W, Wu ZY (2009). Ultrasonic synthesis of the microporous metal-organic framework Cu3(BTC)2 at ambient temperature and pressure: An efficient and environmentally friendly method. Materials Letters.

[59] Qiu C, Wang J, Qin Y, Fan H, Xu X, Jin Z (2018). Green Synthesis of Cyclodextrin-Based Metal-Organic Frameworks through the Seed-Mediated Method for the Encapsulation of Hydrophobic Molecules. J Agric Food Chem.

[60] Liu J, Zhang L, Lei J, Shen H, Ju H (2017). Multifunctional Metal-Organic Framework Nanoprobe for Cathepsin B-Activated Cancer Cell Imaging and Chemo-Photodynamic Therapy. ACS Appl Mater Interfaces.

[61] Lim J, Lee EJ, Choi JS, Jeong NC (2018). Diffusion Control in the *in situ* Synthesis of Iconic Metal-Organic Frameworks within an Ionic Polymer Matrix. ACS Appl Mater Interfaces.

[62] Ranjbar M, Pardakhty A, Amanatfard A, Asadipour A (2018). Efficient drug delivery of beta-estradiol encapsulated in Zn-metal-organic framework nanostructures by microwave-assisted coprecipitation method. Drug Design, Development and Therapy.

[63] Wang T, Li S, Zou Z, Hai L, Yang X, Jia X (2018). A zeolitic imidazolate framework-8-based indocyanine green theranostic agent for infrared fluorescence imaging and photothermal therapy. Journal of Materials Chemistry B.

[64] Mikhaylov G, Klimpel D, Schaschke N, Mikac U, Vizovisek M, Fonovic M (2014). Selective targeting of tumor and stromal cells by a nanocarrier system displaying lipidated cathepsin B inhibitor. Angew Chem Int Ed Engl.

[65] Shen H, Liu J, Lei J, Ju H (2018). A core-shell nanoparticle-peptide@metal-organic framework as pH and enzyme dual-recognition switch for stepwise-responsive imaging in living cells. Chemical Communications.

[66] Gao X, Cui R, Ji G, Liu Z (2018). Size and surface controllable metal-organic frameworks (MOFs) for fluorescence imaging and cancer therapy. Nanoscale.

[67] Liu Y, Hou W, Xia L, Cui C, Wan S, Jiang Y (2018). ZrMOF nanoparticles as quenchers to conjugate DNA aptamers for target-induced bioimaging and photodynamic therapy. Chem Sci.

[68] Chen H, Wang J, Shan D, Chen J, Zhang S, Lu X (2018). Dual-Emitting Fluorescent Metal-Organic Framework Nanocomposites as a Broad-Range pH Sensor for Fluorescence Imaging. Analytical chemistry.

[69] Cheng L, Yang K, Li Y, Zeng X, Shao M, Lee ST (2012). Multifunctional nanoparticles for upconversion luminescence/MR multimodal imaging and magnetically targeted photothermal therapy. Biomaterials.

[70] Deng K, Hou Z, Li X, Li C, Zhang Y, Deng X (2015). Aptamer-mediated up-conversion core/MOF shell nanocomposites for targeted drug delivery and cell imaging. Sci Rep.

[71] Chowdhuri AR, Laha D, Pal S, Karmakar P, Sahu SK (2016). One-pot synthesis of folic acid encapsulated upconversion nanoscale metal organic frameworks for targeting, imaging and pH responsive drug release. Dalton Trans.

[72] Li Y, Gecevicius M, Qiu J (2016). Long persistent phosphors-from fundamentals to applications. Chem Soc Rev.

[73] Sun SK, Wang HF, Yan XP (2018). Engineering Persistent Luminescence Nanoparticles for Biological Applications: From Biosensing/Bioimaging to Theranostics. Accounts of chemical research.

[74] Lv Y, Ding D, Zhuang Y, Feng Y, Shi J, Zhang H (2019). Chromium-Doped Zinc Gallogermanate@Zeolitic Imidazolate Framework-8: A Multifunctional Nanoplatform for Rechargeable *In vivo* Persistent Luminescence Imaging and pH-Responsive Drug Release. Acs Applied Materials & Interfaces.

[75] Richman JS, Moorman JR (2000). Physiological time-series analysis using approximate entropy and sample entropy. American journal of physiology Heart and circulatory physiology.

[76] Sk M, Nandi S, Singh RK, Trivedi V, Biswas S (2018). Selective Sensing of Peroxynitrite by Hf-Based UiO-66-B(OH)2 Metal-Organic Framework: Applicability to Cell Imaging. Inorg Chem.

[77] Yang C, Chen K, Chen M, Hu X, Huan S-Y, Chen L (2019). Nanoscale Metal-Organic Framework Based Two-Photon Sensing Platform for Bioimaging in Live Tissue. Analytical chemistry.

[78] McLeod SM, Robison L, Parigi G, Olszewski A, Drout RJ, Gong X (2020). Maximizing Magnetic Resonance Contrast in Gd(III) Nanoconjugates: Investigation of Proton Relaxation in Zirconium Metal-Organic Frameworks. ACS Appl Mater Interfaces.

[79] Zhang H, Tian XT, Shang Y, Li YH, Yin XB (2018). Theranostic Mn-Porphyrin Metal-Organic Frameworks for Magnetic Resonance Imaging-Guided Nitric Oxide and Photothermal Synergistic Therapy. ACS Appl Mater Interfaces.

[80] Qin L, Sun ZY, Cheng K, Liu SW, Pang JX, Xia LM (2017). Zwitterionic Manganese and Gadolinium Metal-Organic Frameworks as Efficient Contrast Agents for *in vivo* Magnetic Resonance Imaging. ACS Appl Mater Interfaces.

[81] Huang J, Li N, Zhang C, Meng Z (2018). Metal-Organic Framework as a Microreactor for *in situ* Fabrication of Multifunctional Nanocomposites for Photothermal-Chemotherapy of Tumors *in vivo*. ACS Appl Mater Interfaces.

[82] Shu F, Lv D, Song XL, Huang B, Wang C, Yu Y (2018). Fabrication of a hyaluronic acid conjugated metal organic framework for targeted drug delivery and magnetic resonance imaging. RSC Advances.

[83] Ravar F, Saadat E, Gholami M, Dehghankelishadi P, Mahdavi M, Azami S (2016). Hyaluronic acid-coated liposomes for targeted delivery of paclitaxel, in-vitro characterization and in-vivo evaluation. Journal of Controlled Release.

[84] Xu H, Yu N, Zhang J, Wang Z, Geng P, Wen M (2020). Biocompatible Fe-Hematoporphyrin coordination nanoplatforms with efficient sonodynamic-chemo effects on deep-seated tumors. Biomaterials.

[85] Guo C, Xu S, Arshad A, Wang L (2018). A pH-responsive nanoprobe for turn-on F-19-magnetic resonance imaging. Chemical Communications.

[86] Zhang T, Wang L, Ma C, Wang W, Ding J, Liu S (2017). BODIPY-containing nanoscale metal-organic frameworks as contrast agents for computed tomography. Journal of Materials Chemistry B.

[87] Xiong H, Yan J, Cai S, He Q, Wen N, Wang Y (2020). Aptamer-Pyropheophorbide a Conjugates with Tumor Spheroid Targeting and Penetration Abilities for Photodynamic Therapy. Mol Pharm.

[88] Zhang H, Shang Y, Li YH, Sun SK, Yin XB (2019). Smart Metal-Organic Framework-Based Nanoplatforms for Imaging-Guided Precise Chemotherapy. ACS Appl Mater Interfaces.

[89] Su L, Wu Q, Tan L, Huang Z, Fu C, Ren X (2019). High Biocompatible ZIF-8 Coated by ZrO2 for Chemo-microwave Thermal Tumor Synergistic Therapy. Acs Applied Materials & Interfaces.

[90] Tao Y, Sun Y, Shi K, Pei P, Ge F, Yang K (2021). Versatile labeling of multiple radionuclides onto a nanoscale metal-organic framework for tumor imaging and radioisotope therapy. Biomaterials Science.

[91] Zhu W, Yang Y, Jin Q, Chao Y, Tian L, Liu J (2019). Two-dimensional metal-organic-framework as a unique theranostic nano-platform for nuclear imaging and chemo-photodynamic cancer therapy. Nano Research.

[92] Fan Z, Liu H, Xue Y, Lin J, Fu Y, Xia Z (2021). Reversing cold tumors to hot: An immunoadjuvant-functionalized metal-organic framework for multimodal imaging-guided synergistic photo-immunotherapy. Bioact Mater.

[93] Kim J, Piao Y, Hyeon T (2009). Multifunctional nanostructured materials for multimodal imaging, and simultaneous imaging and therapy. Chem Soc Rev.

[94] Wang D, Zhou J, Chen R, Shi R, Zhao G, Xia G (2016). Controllable synthesis of dual-MOFs nanostructures for pH-responsive artemisinin delivery, magnetic resonance and optical dual-model imaging-guided chemo/photothermal combinational cancer therapy. Biomaterials.

[95] Wang GD, Chen H, Tang W, Lee D, Xie J (2016). Gd and Eu Co-Doped Nanoscale Metal-Organic Framework as a T-1-T-2 Dual-Modal Contrast Agent for Magnetic Resonance Imaging. Tomography.

[96] Gao X, Zhai M, Guan W, Liu J, Liu Z, Damirin A (2017). Controllable Synthesis of a Smart Multifunctional Nanoscale Metal-Organic Framework for Magnetic Resonance/Optical Imaging and Targeted Drug Delivery. ACS Appl Mater Interfaces.

[97] Wang D, Zhou J, Shi R, Wu H, Chen R, Duan B (2017). Biodegradable Core-shell Dual-Metal-Organic-Frameworks Nanotheranostic Agent for Multiple Imaging Guided Combination Cancer Therapy. Theranostics.

[98] Xie Z, Cai X, Sun C, Liang S, Shao S, Huang S (2018). O2-Loaded pH-Responsive Multifunctional Nanodrug Carrier for Overcoming Hypoxia and Highly Efficient Chemo-Photodynamic Cancer Therapy. Chem Mater.

[99] Chen L, Zhang J, Zhou X, Yang S, Zhang Q, Wang W (2019). Merging metal organic framework with hollow organosilica nanoparticles as a versatile nanoplatform for cancer theranostics. Acta Biomater.

[100] Mahmoudi M, Serpooshan V, Laurent S (2011). Engineered nanoparticles for biomolecular imaging. Nanoscale.

[101] Tian C, Zhu L, Lin F, Boyes SG (2015). Poly(acrylic acid) Bridged Gadolinium Metal-Organic Framework-Gold Nanoparticle Composites as Contrast Agents for Computed Tomography and Magnetic Resonance Bimodal Imaging. ACS Appl Mater Interfaces.

[102] Li B, Wang X, Chen L, Zhou Y, Dang W, Chang J (2018). Ultrathin Cu-TCPP MOF nanosheets: a new theragnostic nanoplatform with magnetic resonance/near-infrared thermal imaging for synergistic phototherapy of cancers. Theranostics.

[103] Bao J, Zu X, Wang X, Li J, Fan D, Shi Y (2020). Multifunctional Hf/Mn-TCPP Metal-Organic Framework Nanoparticles for Triple-Modality Imaging-Guided PTT/RT Synergistic Cancer Therapy. International Journal of Nanomedicine.

[104] Cai W, Gao H, Chu C, Wang X, Wang J, Zhang P (2017). Engineering Phototheranostic Nanoscale Metal-Organic Frameworks for Multimodal Imaging-Guided Cancer Therapy. ACS Appl Mater Interfaces.

[105] Shang W, Zeng C, Du Y, Hui H, Liang X, Chi C (2017). Core-Shell Gold Nanorod@Metal-Organic Framework Nanoprobes for Multimodality Diagnosis of Glioma. Adv Mater.

[106] Yang P, Men Y, Tian Y, Cao Y, Zhang L, Yao X (2019). Metal-Organic Framework Nanoparticles with NIR Dye for Multimodal-Imaging and Guided Phototherapy. ACS Appl Mater Interfaces.

[107] deKrafft KE, Boyle WS, Burk LM, Zhou OZ, Lin W (2012). Zr- and Hf-based nanoscale metal-organic frameworks as contrast agents for computed tomography. Journal of Materials Chemistry.

[108] Ryu U, Yoo J, Kwon W, Choi KM (2017). Tailoring Nanocrystalline Metal-Organic Frameworks as Fluorescent Dye Carriers for Bioimaging. Inorganic Chemistry.

[109] Gao X, Wang Y, Ji G, Cui R, Liu Z (2018). One-pot synthesis of hierarchical-pore metal-organic frameworks for drug delivery and fluorescent imaging. Crystengcomm.

[110] Yao J, Liu Y, Wang J, Jiang Q, She D, Guo H (2019). On-demand CO release for amplification of chemotherapy by MOF functionalized magnetic carbon nanoparticles with NIR irradiation. Biomaterials.

[111] Zhang Y, Wang L, Liu L, Lin L, Liu F, Xie Z (2018). Engineering Metal-Organic Frameworks for Photoacoustic Imaging-Guided Chemo-/Photothermal Combinational Tumor Therapy. ACS Appl Mater Interfaces.

[112] Zheng X, Wang L, Liu M, Lei P, Liu F, Xie Z (2018). Nanoscale Mixed-Component Metal-Organic Frameworks with Photosensitizer Spatial-Arrangement-Dependent Photochemistry for Multimodal-Imaging-Guided Photothermal Therapy. Chemistry of Materials.

[113] Li PZ, Wang XJ, Zhao Y (2019). Click chemistry as a versatile reaction for construction and modification of metal-organic frameworks. Coordination Chemistry Reviews.

[114] Haddad S, Abánades Lázaro I, Fantham M, Mishra A, Silvestre-Albero J, Osterrieth JWM (2020). Design of a Functionalized Metal-Organic Framework System for Enhanced Targeted Delivery to Mitochondria. Journal of the American Chemical Society.

[115] Dong J, Qiao Z, Pan Y, Peh SB, Yuan YD, Wang Y (2019). Encapsulation and Protection of Ultrathin Two-Dimensional Porous Organic Nanosheets within Biocompatible Metal-Organic Frameworks for Live-Cell Imaging. Chemistry of Materials.

[116] Gao X, Gao Y, Qi R, Han L (2019). One-pot synthesis of a recyclable ratiometric fluorescent probe based on MOFs for turn-on sensing of Mg2+ ions and bioimaging in live cells. New Journal of Chemistry.

[117] Li WY, Yang S, Li YA, Li QY, Guan Q, Dong YB (2019). Synthesis of an MOF-based Hg(2+)-fluorescent probe via stepwise post-synthetic modification in a single-crystal-to-single-crystal fashion and its application in bioimaging. Dalton Trans.

[118] Tabatabaeian K, Simayee M, Fallah-Shojaie A, Mashayekhi F (2019). N-doped carbon nanodots@UiO-66-NH2 as novel nanoparticles for releasing of the bioactive drug, rosmarinic acid and fluorescence imaging. Daru.

[119] Zhang R, Qiao C, Jia Q, Wang Y, Huang H, Chang W (2019). Highly Stable and Long-Circulating Metal-Organic Frameworks Nanoprobes for Sensitive Tumor Detection *In vivo*. Adv Healthc Mater.

[120] Zhou G, Wang YS, Jin Z, Zhao P, Zhang H, Wen Y (2019). Porphyrin-palladium hydride MOF nanoparticles for tumor-targeting photoacoustic imaging-guided hydrogenothermal cancer therapy. Nanoscale Horizons.

[121] Hu X, Lu Y, Zhou L, Chen L, Yao T, Liang S (2020). Post-synthesis strategy to integrate porphyrinic metal-organic frameworks with CuS NPs for synergistic enhanced photo-therapy. J Mater Chem B.

[122] Meng HM, Shi X, Chen J, Gao Y, Qu L, Zhang K (2020). DNA Amplifier-Functionalized Metal-Organic Frameworks for Multiplexed Detection and Imaging of Intracellular mRNA. ACS Sens.

[123] Meng X, Zhang K, Yang F, Dai W, Lu H, Dong H (2020). Biodegradable Metal-Organic Frameworks Power DNAzyme for *in vivo* Temporal-Spatial Control Fluorescence Imaging of Aberrant MicroRNA and Hypoxic Tumor. Anal Chem.

[124] Qin YT, Feng YS, Ma YJ, He XW, Li WY, Zhang YK (2020). Tumor-Sensitive Biodegradable Nanoparticles of Molecularly Imprinted Polymer-Stabilized Fluorescent Zeolitic Imidazolate Framework-8 for Targeted Imaging and Drug Delivery. ACS Appl Mater Interfaces.

[125] Zhang M, Gao Y, Han L, Zhu N, Gao X (2020). The construction of a multifunctional metal-organic framework for targeting tumors and bioimaging. New Journal of Chemistry.

[126] Zheng X, Wang L, Guan Y, Pei Q, Jiang J, Xie Z (2020). Integration of metal-organic framework with a photoactive porous-organic polymer for interface enhanced phototherapy. Biomaterials.

[127] He M, Chen Y, Tao C, Tian Q, An L, Lin J (2019). Mn-Porphyrin-Based Metal-Organic Framework with High Longitudinal Relaxivity for Magnetic Resonance Imaging Guidance and Oxygen Self-Supplementing Photodynamic Therapy. ACS Appl Mater Interfaces.

[128] Jiang Z, Yuan B, Qiu N, Wang Y, Sun L, Wei Z (2019). Manganese-Zeolitic Imidazolate Frameworks-90 with High Blood Circulation Stability for MRI-Guided Tumor Therapy. Nano-Micro Letters.

[129] Liu B, Hu F, Zhang J, Wang C, Li L (2019). A Biomimetic Coordination Nanoplatform for Controlled Encapsulation and Delivery of Drug-Gene Combinations. Angew Chem Int Ed Engl.

[130] Pan Y-B, Wang S, He X, Tang W, Wang J, Shao A (2019). A combination of glioma *in vivo* imaging and *in vivo* drug delivery by metal-organic framework based composite nanoparticles. Journal of Materials Chemistry B.

[131] Nejadshafiee V, Naeimi H, Goliaei B, Bigdeli B, Sadighi A, Dehghani S (2019). Magnetic bio-metal-organic framework nanocomposites decorated with folic acid conjugated chitosan as a promising biocompatible targeted theranostic system for cancer treatment. Mater Sci Eng C Mater Biol Appl.

[132] Tian XT, Cao PP, Zhang H, Li YH, Yin XB (2019). GSH-activated MRI-guided enhanced photodynamic- and chemo-combination therapy with a MnO2-coated porphyrin metal organic framework. Chem Commun (Camb).

[133] Wang D, Wu H, Lim WQ, Phua SZF, Xu P, Chen Q (2019). A Mesoporous Nanoenzyme Derived from Metal-Organic Frameworks with Endogenous Oxygen Generation to Alleviate Tumor Hypoxia for Significantly Enhanced Photodynamic Therapy. Adv Mater.

[134] Wyszogrodzka-Gawel G, Dorozynski P, Giovagnoli S, Strzempek W, Pesta E, Weglarz WP (2019). An Inhalable Theranostic System for Local Tuberculosis Treatment Containing an Isoniazid Loaded Metal Organic Framework Fe-MIL-101-NH2-From Raw MOF to Drug Delivery System. Pharmaceutics.

[135] Zhang SY, Wang ZY, Gao J, Wang K, Gianolio E, Aime S (2019). A Gadolinium(III) Zeolite-like Metal-Organic-Framework-Based Magnetic Resonance Thermometer. Chem.

[136] Chen J, Zhou L, Wang C, Sun Y, Lu Y, Li R (2020). A multifunctional SN38-conjugated nanosystem for defeating myelosuppression and diarrhea induced by irinotecan in esophageal cancer. Nanoscale.

[137] Dehghani S, Hosseini M, Haghgoo S, Changizi V, Akbari Javar H, Khoobi M (2020). Multifunctional MIL-Cur@FC as a theranostic agent for magnetic resonance imaging and targeting drug delivery: *in vitro* and *in vivo* study. J Drug Target.

[138] Ebrahimpour A, Riahi Alam N, Abdolmaleki P, Hajipour-Verdom B, Tirgar F, Ebrahimi T (2020). Magnetic Metal-Organic Framework Based on Zinc and 5-Aminolevulinic Acid: MR Imaging and Brain Tumor Therapy. Journal of Inorganic and Organometallic Polymers and Materials.

[139] Hamideh RA, Akbari B, Fathi P, Misra SK, Sutrisno A, Lam F (2020). Biodegradable MRI Visible Drug Eluting Stent Reinforced by Metal Organic Frameworks. Adv Healthc Mater.

[140] Shang W, Peng L, Guo P, Hui H, Yang X, Tian J (2020). Metal-Organic Frameworks as a Theranostic Nanoplatform for Combinatorial Chemophotothermal Therapy Adapted to Different Administration. ACS Biomater Sci Eng.

[141] Wang Z, Yu W, Yu N, Li X, Feng Y, Geng P (2020). Construction of CuS@Fe-MOF nanoplatforms for MRI-guided synergistic photothermal-chemo therapy of tumors. Chemical Engineering Journal.

[142] Li S, Shi X, Wang H, Xiao L (2021). A multifunctional dual-shell magnetic nanocomposite with near-infrared light response for synergistic chemo-thermal tumor therapy. J Biomed Mater Res B Appl Biomater.

[143] Liu X, Zhu X, Qi X, Meng X, Xu K (2021). Co-Administration of iRGD with Sorafenib-Loaded Iron-Based Metal-Organic Framework as a Targeted Ferroptosis Agent for Liver Cancer Therapy. Int J Nanomedicine.

[144] Meng Z, Huang H, Huang D, Zhang F, Mi P (2021). Functional metal-organic framework-based nanocarriers for accurate magnetic resonance imaging and effective eradication of breast tumor and lung metastasis. J Colloid Interface Sci.

[145] Zhou H, Qi M, Shao J, Li X, Zhou Z, Yang S (2021). Tumor micro-environment sensitive 19F-magnetic resonance imaging *in vivo*. Journal of Magnetism and Magnetic Materials.

[146] Zhu Y, Xin N, Qiao Z, Chen S, Zeng L, Zhang Y (2020). Bioactive MOFs Based Theranostic Agent for Highly Effective Combination of Multimodal Imaging and Chemo-Phototherapy. Advanced Healthcare Materials.

[147] Wang Z, Liu B, Sun Q, Feng L, He F, Yang P (2021). Upconverted Metal-Organic Framework Janus Architecture for Near-Infrared and Ultrasound Co-Enhanced High Performance Tumor Therapy. ACS Nano.

[148] Pu Y, Zhu Y, Qiao Z, Xin N, Chen S, Sun J (2021). A Gd-doped polydopamine (PDA)-based theranostic nanoplatform as a strong MR/PA dual-modal imaging agent for PTT/PDT synergistic therapy. J Mater Chem B.

[149] Gao X, Zhang M, Lv L (2021). One-pot synthesis of multifunctional metal-organic frameworks for magnetic resonance/optical imaging. Materials Letters.

[150] Cheng Y, Wen C, Sun YQ, Yu H, Yin XB (2021). Mixed-Metal MOF-Derived Hollow Porous Nanocomposite for Trimodality Imaging Guided Reactive Oxygen Species-Augmented Synergistic Therapy. Advanced Functional Materials.

[151] Xu M, Chi B, Han Z, He Y, Tian F, Xu Z (2020). Controllable synthesis of rare earth (Gd3+,Tm3+) doped Prussian blue for multimode imaging guided synergistic treatment. Dalton Transactions.

[152] Wang Z, Liu B, Sun Q, Dong S, Kuang Y, Dong Y (2020). Fusiform-Like Copper(II)-Based Metal-Organic Framework through Relief Hypoxia and GSH-Depletion Co-Enhanced Starvation and Chemodynamic Synergetic Cancer Therapy. ACS Appl Mater Interfaces.

[153] Ling D, Li H, Xi W, Wang Z, Bednarkiewicz A, Dibaba ST (2020). Heterodimers made of metal-organic frameworks and upconversion nanoparticles for bioimaging and pH-responsive dual-drug delivery. J Mater Chem B.

[154] Guo H, Xia Y, Feng K, Qu X, Zhang C, Wan F (2020). Surface Engineering of Metal-Organic Framework as pH-/NIR-Responsive Nanocarrier for Imaging-Guided Chemo-Photothermal Therapy. Int J Nanomedicine.

[155] Zhang D, Ye Z, Wei L, Luo H, Xiao L (2019). Cell Membrane-Coated Porphyrin Metal-Organic Frameworks for Cancer Cell Targeting and O2-Evolving Photodynamic Therapy. ACS Appl Mater Interfaces.

[156] Lin C, Chi B, Xu C, Zhang C, Tian F, Xu Z (2019). Multifunctional drug carrier on the basis of 3d-4f Fe/La-MOFs for drug delivery and dual-mode imaging. Journal of Materials Chemistry B.

[157] Gao X, Cui R, Song L, Liu Z (2019). Hollow structural metal-organic frameworks exhibit high drug loading capacity, targeted delivery and magnetic resonance/optical multimodal imaging. Dalton Trans.

[158] Chen Y, Liu W, Shang Y, Cao P, Cui J, Li Z (2019). Folic acid-nanoscale gadolinium-porphyrin metal-organic frameworks: fluorescence and magnetic resonance dual-modality imaging and photodynamic therapy in hepatocellular carcinoma. Int J Nanomedicine.

[159] Pinter NK, Klein JP, Mechtler LL (2016). Potential Safety Issues Related to the Use of Gadolinium-based Contrast Agents. Continuum (Minneapolis, Minn).

[160] Gulani V, Calamante F, Shellock FG, Kanal E, Reeder SB (2017). Gadolinium deposition in the brain: summary of evidence and recommendations. The Lancet Neurology.

